# Identifying Novel Cell Cycle Proteins in Apicomplexa Parasites through Co-Expression Decision Analysis

**DOI:** 10.1371/journal.pone.0097625

**Published:** 2014-05-19

**Authors:** Carrie L. Butler, Olivier Lucas, Stefan Wuchty, Bin Xue, Vladimir N. Uversky, Michael White

**Affiliations:** 1 Department of Molecular Medicine, Morsani College of Medicine, University of South Florida, Tampa, Florida, United States of America; 2 Department of Global Health, College of Public Health, University of South Florida, Tampa, Florida, United States of America; 3 Florida Center for Drug Discovery and Innovation, University of South Florida, Tampa, Florida, United States of America; 4 National Center for Biotechnology Information, National Institutes of Health, Bethesda, Maryland, United States of America; University of Wisconsin Medical School, United States of America

## Abstract

Hypothetical proteins comprise roughly half of the predicted gene complement of *Toxoplasma gondii* and *Plasmodium falciparum* and represent the largest class of uniquely functioning proteins in these parasites. Following the idea that functional relationships can be informed by the timing of gene expression, we devised a strategy to identify the core set of apicomplexan cell division cycling genes with important roles in parasite division, which includes many uncharacterized proteins. We assembled an expanded list of orthologs from the *T. gondii* and *P. falciparum* genome sequences (2781 putative orthologs), compared their mRNA profiles during synchronous replication, and sorted the resulting set of dual cell cycle regulated orthologs (744 total) into protein pairs conserved across many eukaryotic families versus those unique to the Apicomplexa. The analysis identified more than 100 ortholog gene pairs with unknown function in *T. gondii* and *P. falciparum* that displayed co-conserved mRNA abundance, dynamics of cyclical expression and similar peak timing that spanned the complete division cycle in each parasite. The unknown cyclical mRNAs encoded a diverse set of proteins with a wide range of mass and showed a remarkable conservation in the internal organization of ordered versus disordered structural domains. A representative sample of cyclical unknown genes (16 total) was epitope tagged in *T. gondii* tachyzoites yielding the discovery of new protein constituents of the parasite inner membrane complex, key mitotic structures and invasion organelles. These results demonstrate the utility of using gene expression timing and dynamic profile to identify proteins with unique roles in Apicomplexa biology.

## Introduction

Apicomplexa are a phylum of unicellular, obligate intracellular parasites that includes pathogens of medical and veterinary importance. Significant human diseases such as toxoplasmosis caused by *Toxoplasma gondii* and the deadliest form of malaria, caused by *Plasmodium falciparum* are two examples. The rapid asexual growth of parasites resulting in the cellular lysis and inflammation is at the center of these diseases, where the burden of growth is paramount in disease pathology [1], [2] and drug resistance is a constant concern. Therefore, understanding the mechanisms responsible for growth and invasion of these parasites is important for discovering new therapeutic targets and maintaining an active pipeline of new clinical treatments.

Apicomplexa parasites have evolved unique and effective strategies for intracellular replication. *T. gondii* and *P. falciparum* diverged several hundred million years ago [3] and represent two modern endpoints of the apicomplexan evolution (coccidia and haemosporida families). The *T. gondii* tachyzoite stage and the *P. falciparum* merozoite growing in human red blood cells are the major Apicomplexa model organisms for which growth synchrony models are robust [4], [5] and advanced molecular genetics can be performed. *T. gondii* tachyzoites undergo endodyogeny where binary division produces two internal daughters within the mother cell [6]. *P. falciparum* merozoites undergo schizogony, where multiple rounds of nuclear replication are followed by parasite budding to produce infectious parasites [7]. Endodyogeny and schizogony are similar in that they both produce new daughter parasites internally, which consumes the mother cell, and each division cycle (∼48 h for *P. falciparum* and ∼8 h for *T. gondii*) consists of a single major G1 period followed by one or more S/M phases concluding with concerted cytokinesis.

The sequencing of the *P. falciparum* genome in 2002 was the beginning of genomics for parasites of this phylum [8] and was quickly followed by the first insights into the functional genomics of the *P. falciparum* intraerythrocytic cell cycle the next year [9]. One of the major insights of this first transcriptome effort introduced the concept of “just-in-time” delivery of proteins during the parasite division cycle. Remarkably, all cyclical mRNAs (∼40% of transcripts) showed single peak expression that unfolds in a progressive cascade across the 48 h intraerythrocytic cycle [9]. We confirmed that this elaborate cascade also occurs in the distantly related apicomplexan *T. gondii* [10]. The relatively simple binary division of the *T. gondii* tachyzoite also revealed that cell cycle transcription unfolds in two major waves where mRNAs encoding ancient genes that include many DNA replication, transcription and translation genes show maximum expression levels in the G1 phase, while the unique apicomplexan genes involved in building invasion and internal daughter structures peak in the S/M phases [10]. While these waves are not as apparent in *P. falciparum* cell cycle transcription [9] the order of mRNA expression follows a similar evolutionary segregation of cell cycle timing [10].

The peculiar cell division mechanisms observed in apicomplexans that occur in the S/M/C phase of the cell cycle [11] involve many apicomplexan genes that have unknown functions. The overwhelming number of unknown proteins in these parasites (38-58%) is a challenge, as they do not have recognizable domains, making it difficult to accurately predict their function. Originally designated as hypothetical gene products in Apicomplexa parasites many of these proteins are now identified as expressed proteins through multiple high throughput proteomics surveys [12], [13]. Few studies have employed a strategy to experimentally investigate unknown proteins on a large scale leaving principally the computationally approaches the task of assigning predicted function [14], [15], [16], [17]. The major drawback to *in silico* approaches is reliance on known processes in other cells. An accepted strategy to characterize unknown proteins is to establish some level of guilt by association (similar expression profile or protein-protein interaction from global interactome). Here we demonstrate a functionally unbiased approach based on cell cycle co-expression with no prerequisite of function that would enable us to identify new proteins involved in parasite division. Through this approach we have defined the core cell division cycling (CDC) genes conserved in *T. gondii* and *P. falciparum*. A selection of unknown CDC genes was tagged in the *T. gondii* tachyzoite stage, revealing novel unknown proteins are housed in nearly every subcellular compartment of the highly organized tachyzoite cell.

## Results

### Identifying cell division cycling (CDC) genes conserved in divergent Apicomplexa asexual stages

There are 6,372 predicted protein-coding genes in the *P. falciparum* genome with 2,432 (38.2%) of these genes (strain HB3, www.plasmodb.org, V8.0) encoding unique family proteins many of which have no ortholog outside the Apicomplexa phylum (many are designated hypothetical or putative proteins). A similar genetic landscape characterizes *T. gondii* (strain ME49, www.toxodb.org, toxoDB V7.1) genome sequence where computer annotation predicts 8,102 genes. Here an even higher occurrence of hypothetical proteins is annotated (58.2%, n = 4,717 in ME49 strain). These large groups of proteins likely hold the key to important parasite biology, yet a basic challenge is to know which unknown proteins should be investigated in order to uncover important new biology. We hypothesized that defined mRNA co-expression could provide a rationale for making some of the tough experimental choices. The assembly of new infectious parasites in *P. falciparum* and *T. gondii* is directed by an ordered cell cycle transcriptome that delivers proteins in a “just-in-time” sequence [9], [10]. Each cyclical transcript reaches a peak once per cycle whether the cell cycle length is 8 h in *T. gondii* tachyzoites or 48 h in the *P. falciparum* merozoites. In *T. gondii*, the timing of cell cycle transcripts is organized in two waves that separate mRNA peak expression in two distinct G1 and S/M/C subtranscriptomes [10].

**Figure 1:**
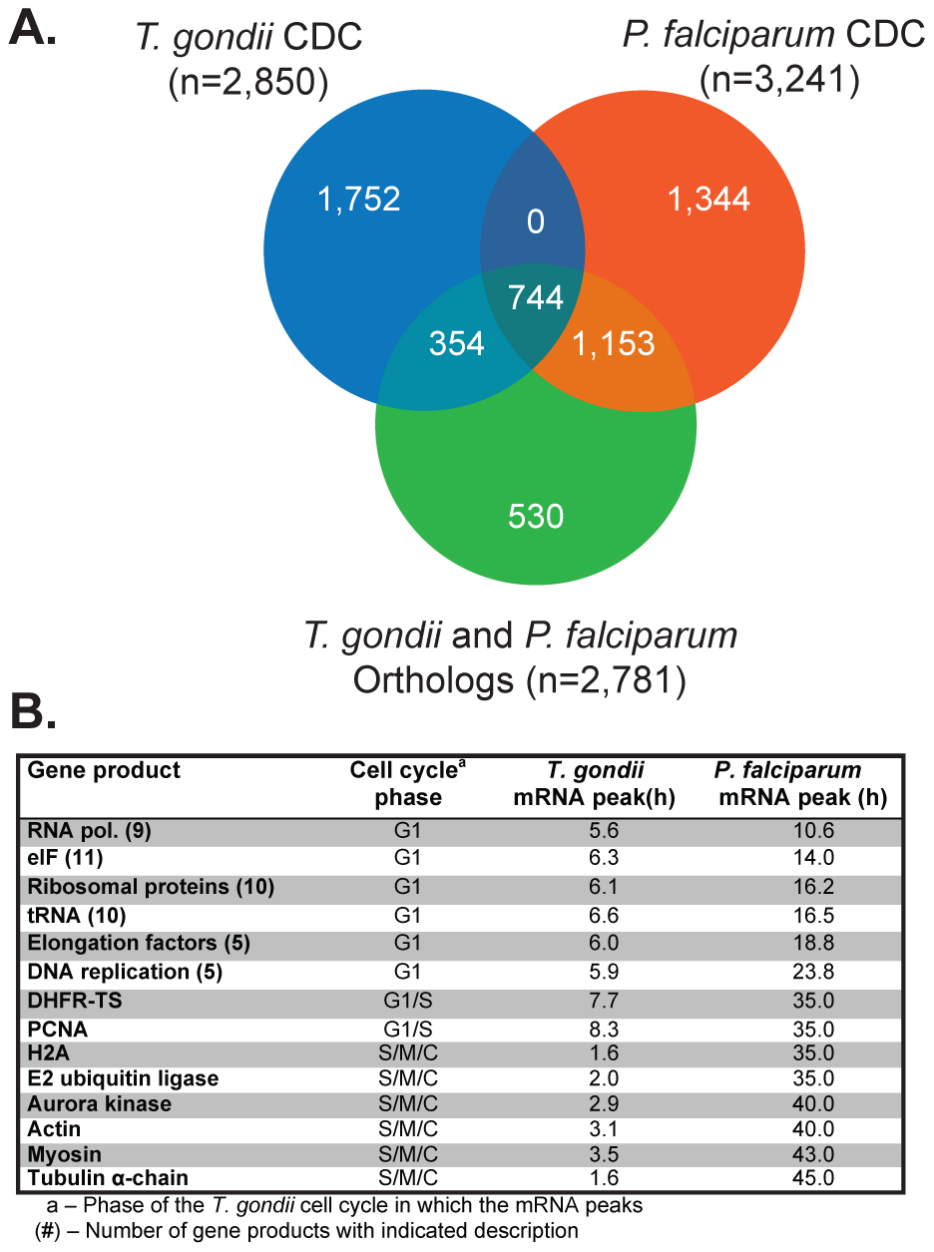
Identifying conserved Apicomplexa cell division cycling (CDC) genes of *T. gondii* and *P. falciparum*. [**A**.] The Venn diagram indicates the intersections between orthologous protein pairs and sets of cell cycle regulated mRNAs in *T. gondii* and *P. falciparum* asexual stages. A total of 744 mRNAs encoding *T. gondii* and *P. falciparum* orthologs have cyclical profiles in replicating merozoites and tachyzoites. Other gene sets obtained from this analysis (see Dataset S1) includes ortholog mRNAs exclusively cell cycle regulated in each species (354 for *T. gondii* and 1,153 for *P. falciparum*) and cyclical mRNAs that do not encode ortholog proteins (1,752 for *T. gondii* and 1,344 for *P. falciparum*). The 530 orthologs that are not cell cycle regulated were excluded from this study. [**B**.] Among the set of 744 orthologs that are dual cell cycle regulated (drCDC genes) are mRNAs encoding well-known eukaryotic proteins. For example, factors involved in transcription and translation machineries (*e.g*. RNA polymerase, ribosomal proteins, etc.) typically peak in G1, while mRNAs encoding cytoskeletal genes (*e.g*. actin, myosin, and tubulin) peak in the S phase and mitosis.

We exploited this remarkable shared sequence of gene expression by incorporating this information into a decision tree with three principle binary choices: step 1) *T. gondii* and *P. falciparum* orthologs or not, step 2) conserved cyclical mRNA or not, and step 3) novel protein or not. We performed this analysis by first defining conserved ortholog protein-coding genes in *T. gondii* and *P. falciparum* using an expanded list (see Materials and Methods) that employed modified parameters allowing orthologs to be called based on more limited regions of conserved protein topology and not restricted by length. This process yielded 2,781 orthologous pairs from the complete set of *T. gondii* and *P. falciparum* predicted genes (see Fig. 1A and Dataset S1). In the next step we determined how many orthologs were encoded by a periodic transcript. The cell cycle transcriptomes utilized were the 3,241 cyclical transcripts in the *P. falciparum* HB3 strain (synchronized by two consecutive sorbitol treatments for three generations, for a total of six treatments) that have one peak of maximum expression with an amplitude >1.5 [9], and the 2,850 cubic b-spline modeling of mRNAs expressed in synchronized *T. gondii* RH^TK+^ tachyzoites [10]. This second step in our decision tree identified 744 orthologs that encode dual regulated cell division cycling (drCDC) mRNAs (Fig. 1A). This analysis also identified 354 orthologs that encoded CDC mRNAs in *T. gondii*, but not in *P. falciparum*, and conversely 1,153 orthologs had the reverse relationship (*i.e*. *P. falciparum* CDC, not CDC in *T. gondii*; see Dataset S1 for all gene lists and annotations). There were few notable gene clusters in the 354 CDC *T. gondii* orthologs that are constitutive in *P. falciparum* (see Dataset S1). The orthologs expressed by CDC transcripts only present in *P. falciparum* encoded for many ribosomal and histone proteins, which was first noted to be constitutive in *T. gondii* [10]. It is possible the *P. falciparum* synchrony model is more sensitive to changes in mRNA due to its longer length and frequent sampling (46 hours of the 48 h intraerythrocytic cycle), or alternatively the scale of new parasites produced by the *P. falciparum* merozoites influences this difference. A single *P. falciparum* merozoite division cycle produces 16–26 new parasites compared to the simple 2-fold production of daughter parasites in the *T. gondii* tachyzoite cell cycle. By default CDC genes encoding non-orthologs were also identified here and are associated with important biology observed in these parasites (Dataset S1). The 1,344 CDC genes exclusive to *P. falciparum* included merozoite surface proteins, *P. falciparum* export proteins, and VAR and rifin (RIF) proteins involved in antigen variation for which there is no equivalent mechanism in *T. gondii*. Similarly, the 1,752 *T. gondii*-specific CDC genes include surface antigens and SAG1-related sequence (SRS) domain proteins as well as secreted proteins required for *T. gondii* invasion that are housed in the apical microneme and rhoptry organelles, which is consistent with the unique compositions of apical organelles responsible for transmission in unique host cell environments [18], [19].

**Figure 2:**
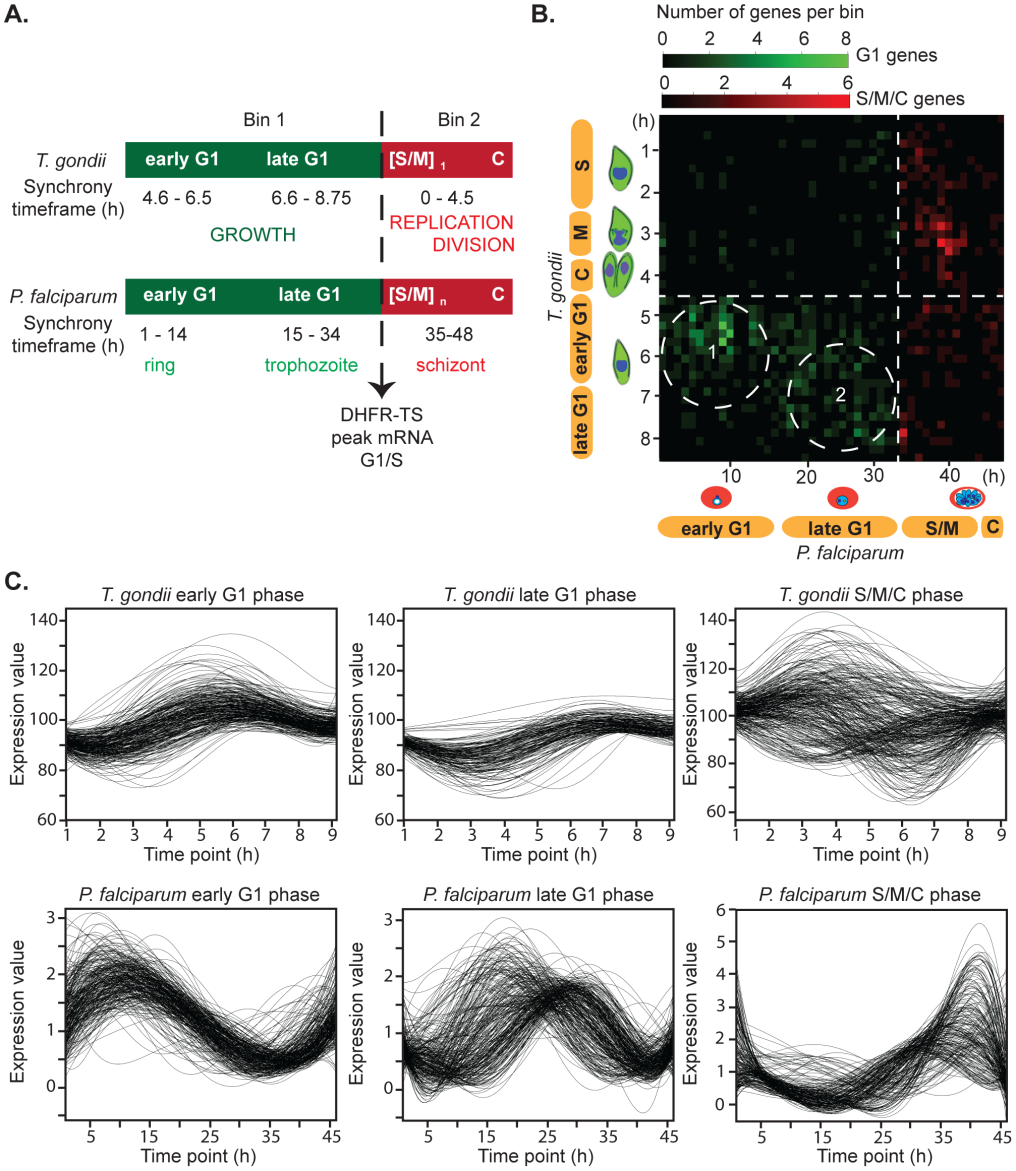
The timing of core drCDC genes defines clusters of co-regulated mRNAs. [**A**.] Cell cycle transcriptome data from *T. gondii* [10] and *P. falciparum* [9] asexual stages were analyzed, allowing the alignment of the cell cycles by the peak timing of canonical drCDC mRNAs as exemplified by DHFR-TS (dashed line). In *T. gondii*, tachyzoite replication involves single S and mitotic [S/M] phases followed by parasite budding (cytokinesis = C). In the *P. falciparum* merozoite repeated rounds of chromosome replication and nuclear division produce multiple merozoites from a single infection. Despite these scale differences in replication, adopting this procedure generates a basic partition of the cell cycles: Bin 1 encompasses mRNAs that peak in G1, while Bin 2 covers mRNAs with maximum expression in S phase, mitosis and cytokinesis. Further sorting of G1 was apparent by the self-clustering of the peak timing of mRNAs encoding transcription and translation (early G1) from those mRNAs encoding known DNA replication factors (late G1): early G1 of *T. gondii* = 4.6–6.5 h, *P. falciparum* = 1–14 h; late G1 for *T*. gondii = 6.6–8.75 h, *P*. falciparum = 15–34 h; and S/M/C of *T. gondii* = 0–4.5 h, *P. falciparum* = 35–48 h. [**B**.] Pairwise expression timing of all 744 drCDC mRNAs using the alignment scheme of [A.]. The G1/S transition (dashed line) and matched G1 timing is plotted in the lower left quadrant, while matched S/M/C timing corresponds to the upper right quadrant. The relatively few mRNAs indicated in the upper left and lower right quadrants represent drCDC gene pairs with mismatched mRNA timing. Color intensity (green: peak in G1, red: peak in S/M/C) indicates the increase in the number of genes showing similar timing in each cell cycle. In the G1 phase, two clusters correspond to the peak timing of mRNAs. The cluster of paired mRNAs in circle 1 correspond to many general transcription/translation genes, while in circle 2 co-expressed mRNAs are enriched for DNA synthetic factors. [**C**.] Individual mRNA profiles for co-expressed mRNAs that peak in early G1, late G1 and S/M/C phases are displayed.

The 744 drCDC mRNAs encoding orthologous protein pairs dual regulated in *P. falciparum* and *T. gondii* included well-recognized cell cycle factors such as dihydrofolate reductase thymidylate synthase (DHFR-TS) and DNA and RNA polymerases (see Fig. 1B for a partial list of canonical drCDC genes). The peak timing of these canonical drCDC mRNAs are conserved in many eukaryotes with transcription/translation and DNA replication genes having peak expression in the G1 phase, and genes encoding cytoskeletal structural proteins such as actin and tubulin peaking during S/M/C phase of the cell cycles. The relative timing of canonical drCDC mRNAs provided reference points to align the synchrony models of *T. gondii* RH^TK+^ tachyzoites (8.75 h cycle length) [4], [10] and sorbitol-treated merozoites of *P. falciparum* (48 h cycle length) [5]. The two parasite cell cycles were aligned here at the G1/S transition by DHFR-TS mRNA peak expression and generated two basic bins of G1 (bin 1) versus S/M/C mRNA timing (bin 2) (Fig. 2A). Due to the method of synchrony that arrests RH^TK+^ tachyzoites in late G1/early-S transition (thymidine block and release) [4], [10], the *T. gondii* G1 (bin 1) corresponds to the 4.6–8.75 h post-thymidine release, while the S/M/C (bin 2) is 0–4.5 h post-thymidine release. The *P. falciparum* synchrony method enriches for the beginning ring stage, and thus, the G1 (bin 1) is 0–34 h post-infection and the 35–48 h post-infection time points represent parasites in S/M/C (bin 2). Further partition of G1 was accomplished by aligning the peak timing of mRNAs encoding transcription and translation (early G1) from those mRNAs encoding known DNA replication factors (late G1) in *T. gondii* and *P. falciparum*. Early G1 for *T. gondii* was defined as 4.6–6.5 h and 1–14 h for *P. falciparum*; late G1 for *T*. gondii was defined as 6.6–8.75 h and 15–34 h for *P. falciparum*. To ensure that drCDC genes encoded mRNAs with conserved cell cycle timing, we generated a data matrix based on each pair of orthologous drCDC genes (744 total) and their peak mRNA expression timing was aligned to the matched cell cycles (Fig. 2B). We summed the number of ortholog pairs sharing the same peak mRNA timing and represented the total number of mRNAs with similar peak expression in both species as color intensity on the linear gradient (see color legend Fig. 2B). The bottom left quadrant of this matrix graph corresponds to drCDC orthologs with maximum expression in the G1 phase. As noted in earlier studies [10] transcripts that peak in the first half of G1 are enriched for proteins involved in transcriptional and translational mechanisms (Fig. 2B, dashed circle 1), while late G1 is characterized by the expression of genes required for chromosome replication (Fig. 2B, dashed circle 2). This profile is conserved in many eukaryotes and both *T. gondii* and *P. falciparum* follow this pattern. The top right graph quadrant corresponds to mRNAs with cyclical profiles that peak in the S/M/C phases. The specific patterns of co-expression for each of these mRNA subsets are shown in the full expression profiles across the synchronous growth timeframes (Fig. 2C). Distinct co-expression profiles characterize each mRNA group, as can be observed in *P. falciparum* and *T. gondii* genes. In *T. gondii*, G1 mRNAs are much less dynamic than their *P. falciparum* counterparts. As with the ribosomal and histone genes above, this difference is not fully understood, but could be related to the different biotic production of each division cycle. The *P. falciparum* S/M/C mRNAs displayed tight peak expression compared to S/M/C *T. gondii* mRNAs. Here there were two distinct *T. gondii* patterns, the majority of mRNAs peaked around 3 h, while a minor population was maximum at the G1/S boundary (8.75 h time point) and declined immediately post-thymidine release. Generally mRNA expression for asexual stage apicomplexans correlates well with the peak of the encoded protein [9], [20]. Altogether, these results demonstrated that the mRNA profiles as well as the peak times were shared in each cell cycle class indicating that both timing and amplitude are conserved.

### A large class of dual-CDC genes encodes novel cell cycle proteins

In the third sorting step, we categorized the 744 drCDC genes (Dataset S1) as novel or not, which generated a list of 125 genes annotated in EupathDB as “hypothetical” and were designated here as drCDC unknowns (drCDC-UNK). Employing an expanded list of putative orthologs captured a larger number of drCDC-UNK genes than is possible with other approaches to orthology assignment such as OrthoMCL, which use a more restricted protein length criterion. For example, only 84 (66%) of the 125 *P. falciparum* novel proteins identified here as drCDC-UNKs, would have been classified using OrthoMCL in step 1 (eupathdb.org, v.2.12). We expected that drCDC-UNK gene lists would be enriched for phylum-specific proteins that could have roles in the apicoplast, invasion and unique cell division functions since these processes are specialized in the Apicomplexa. The evolutionary specificity was confirmed as 109 of the drCDC-UNK were found only in genome sequence of Apicomplexa parasites, while only 16 drCDC-UNK genes had wider conservation including a few orthologs shared with human cells. The drCDC-UNK genes conserved outside the Apicomplexa phylum are interesting and may represent undiscovered cell cycle factors present in the original eukaryote. A correlation between evolutionary distribution and essentiality has been noted [21] and the recent discovery of a novel splicing factor in *Toxoplasma* that is widely conserved across the eukaryotes [22] also highlights these possibilities.

**Figure 3:**
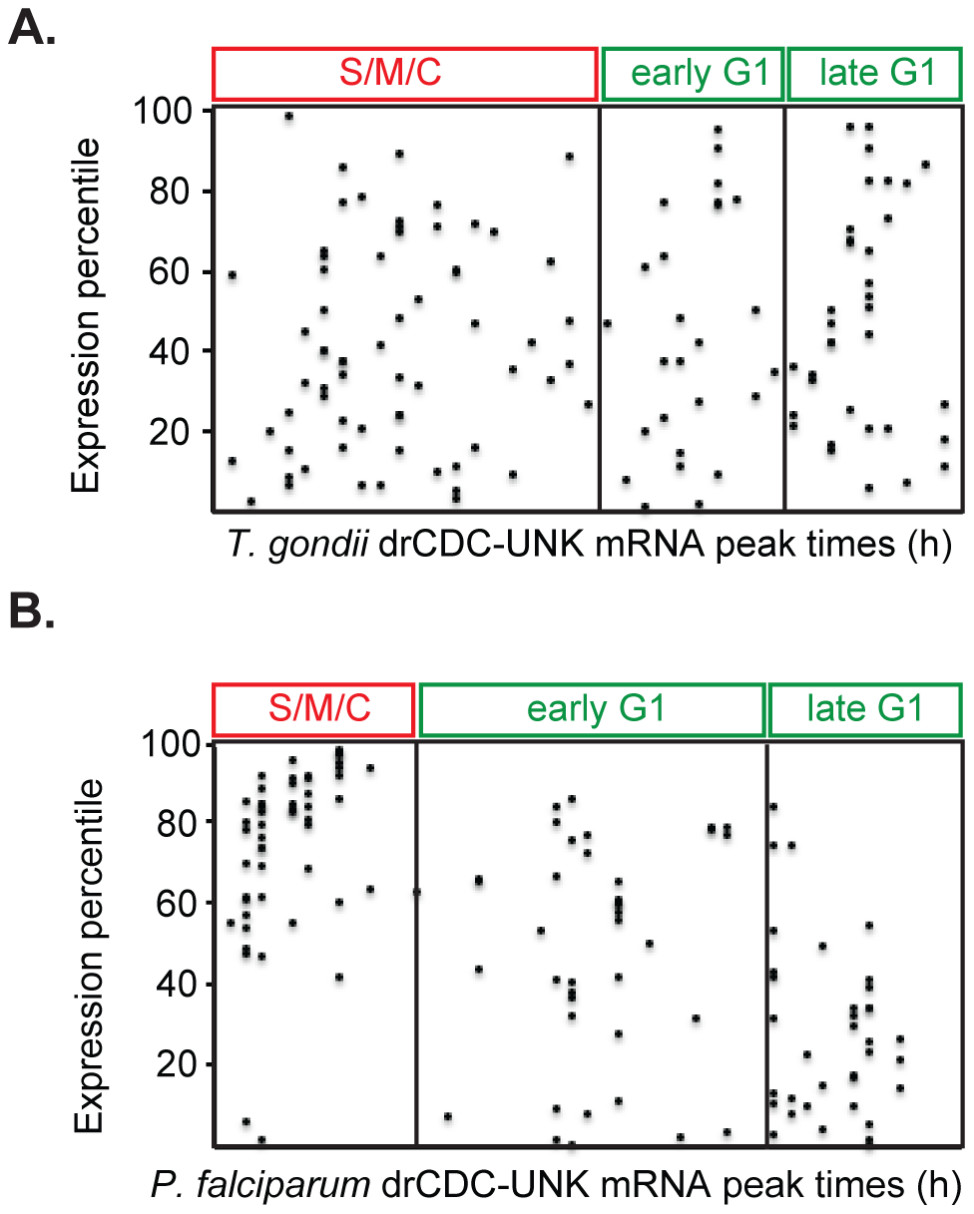
The peak expression of drCDC mRNAs encoding novel proteins is distributed throughout the parasite cell division cycles. The peak time of expression for 125 drCDC-UNK mRNAs (x-axis) in synchronized *T. gondii* tachyzoites [A.] and *P. falciparum* merozoites [B.] are shown. Note that these mRNAs encompass a large range of expression levels (relative expression percentile, y-axis) from low to highly abundant. The exception was the uniformly abundant drCDC-UNK mRNAs of *P. falciparum* merozoites with maximum levels in the S/M/C periods.

Published half-life profiles for the *P. falciparum* drCDC-UNK mRNAs were analyzed with respect to the steady-state peak times [20]. Most of the mRNAs (89% or n = 113) had the highest half-life at or within a few hours following the timeframe of the maximum mRNA expression in the cell cycle (Fig. S1). The most abundant category (n = 36) was for mRNAs peaking in schizonts (31–44 h post infection), for which the longest half-life was in late schizonts (45–48 h post infection). Thus, steady-state mRNA peak timing is likely a reasonable indicator for when transcriptional inputs yield their greatest influences on protein expression in the parasite cell cycle. Analyzing relative abundance of the drCDC-UNK mRNAs in the context of timing of peak expression (Fig. 3A, B) reveals that in *T. gondii* and *P. falciparum* novel drCDC-UNK mRNAs spanned all abundance classes and were expressed at all time points throughout the cell cycle. Interestingly, the cluster of drCDC-UNK mRNAs expressed in the second half of the *P. falciparum* merozoite cell cycle were all highly abundant transcripts (Fig. 3B) possibly reflecting the scaling difference between these two division processes (*T. gondii* division is 2x, while *P. falciparum* is ∼10x) as was mentioned earlier.

### Novel Apicomplexa drCDC-UNK proteins have conserved intrinsic disorder propensity

**Figure 4:**
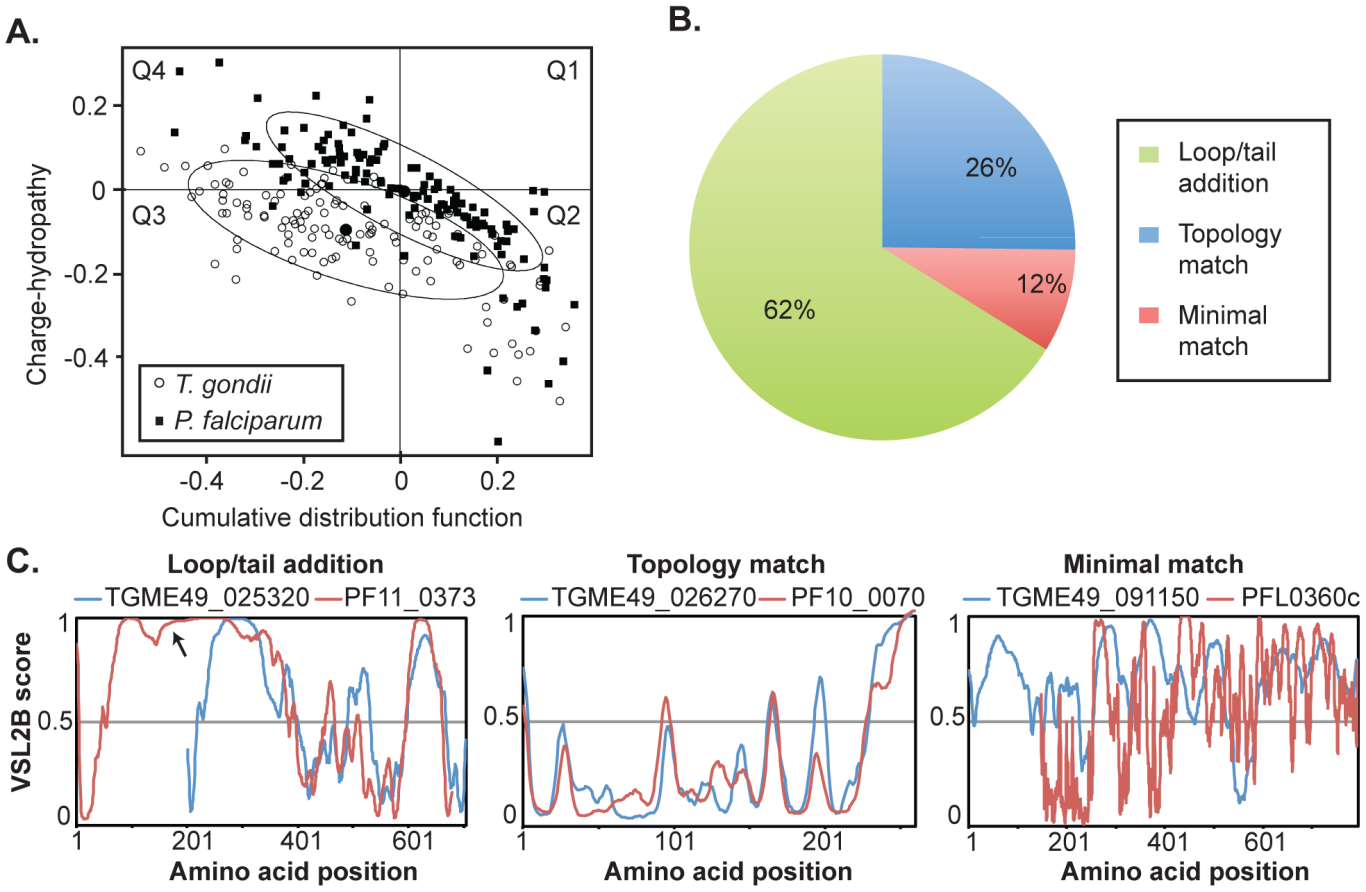
Novel drCDC-UNK proteins have highly conserved structural features. [**A**.] CH-CDF analysis of the drCDC-UNK proteins. Here, the coordinates of each point were calculated as a distance of the corresponding protein in the CH-plot from the boundary (Y-coordinate) and an average distance of the respective CDF curve from the CDF boundary (X-coordinate). The four quadrants correspond to the following predictions: Q1 (upper right), proteins predicted to be disordered by CH-plots (positive values), but ordered by CDFs (negative values); Q2, ordered proteins; Q3, proteins predicted to be disordered by CDFs, but compact by CH-plots (i.e., putative molten globules or mixed proteins); Q4, proteins predicted to be disordered by both methods (i.e., proteins with extended disorder). This plot shows that roughly half of the 125 drCDC-UNK proteins are expected to be disordered as a whole with the degree of disorder slightly higher in *T. gondii*. The ellipses encompass 80% of the data points for each species: *P. falciparum* = closed squares, *T. gondii* = open circles. [**B. and C**.] The 125 drCDC-UNK pairwise disorder/order alignments sorted into three general categories. An example of an ortholog pair displaying loop/tail additions, compared to topologies that closely match versus a pair that minimally matched. Order/disorder plots (VSL2B scores, >0.5 = disordered) for *T. gondii* (blue) versus *P. falciparum* (red) proteins were aligned by best-fit methods. Pairwise graphs for all 125 drCDC-UNK proteins are included in Supplemental Information.

To further understand drCDC-UNK proteins from *T. gondii* and *P. falciparum*, we analyzed their intrinsic disorder propensities for each orthologous pair in order to explore whether the disorder/order profiles were conserved over a long evolutionary time. These modern parasites represent deep branches in the Apicomplexa lineage and have lost all chromosome syntenic structure (i.e. no three genes are in the same order) [23]. Since evolution typically conserves protein folding [21] and predisposition for functionally important intrinsic disorder [24], [25], [26], [27], these features can aid in identifying and verifying orthologous relationships where primary amino acid sequence does not reveal ancestral linkage with significant confidence [21], [24], [25], [26], [27]. The drCDC-UNK protein set comprises a range of predicted protein masses (8 to >1,000 kDa) with wide variation in predicted disorder based on charge/hydrophobicity scores, which is a binary classifier of protein disorder at the whole protein scale. In *T. gondii*, 76 (60%) of the drCDC-UNK proteins were predicted to be disordered (Fig. 4A, left two quadrants, open circles), while in *P. falciparum* 54 (43%) are disordered (Fig. 4A, left two quadrants, closed squares).

The overall molecule-level disorder propensity defined from average charge/hydrophobicity values for each protein does not reveal how evolutionary selection has operated to shape protein topology on a per amino acid scale. We examined this question for each drCDC-UNK protein pair by analyzing per-residue disorder prediction. Sequence distributions of the PONDR VSL2B scores (>0.5 indicates disorder) were analyzed for each protein pair as previously described [28] and profiles of the predicted intrinsic disorder propensity were compared by best-fit criteria and independent of order/disorder for the drCDC-UNK proteins. The pairwise matching of the VSL2B profiles revealed three broad categories of protein alignment (Fig. 4C): (i) highly conserved intrinsic order/disorder profiles (n = 32, see all pairings in Fig. S2–3), (ii) similar profiles with obvious tail(s) or domain(s)/loop(s) insertions or complex additions (n = 78, see all in Fig. S4–7) and (iii) minimal profile matches of both ordered and disordered proteins (n = 15, see all in Fig. S8). Remarkably the drCDC-UNK matched protein pairs include examples of proteins from both ends of the order/disorder spectrum with some fully ordered (5/32) and others nearly completely disordered (3/32) indicating that order/disorder itself is not the driving evolutionary characteristic (see Fig. S2–3). However, when domains have evolved that are unique to either parasite species the addition is typically intrinsically disordered. These additions occur at the N or C-terminus tail or display complex additions of various combinations of additions with some internal loops. In the set of *T. gondii* drCDC-UNK proteins there were 14 N-terminal and 8 C-terminal additions. Similar changes were observed in *P. falciparum* with 8 N-terminal and 7 C-terminal mostly disordered tails added. Complex additions involving a combination of additions and loops were observed for both *T. gondii* and *P. falciparum* drCDC-UNK proteins (n = 41). The abundant class of protein topology profiles with loop/tail additions is represented by TGME49_025320 for *T. gondii* and PF11_0373 for *P. falciparum* where the *P. falciparum* protein extended the loop region (indicated by an arrow) in the N-terminus (Fig. 4C Loop/tail addition). An example of matching protein topology is the pair of proteins, *T. gondii* TGME49_026270 and *P. falciparum* PF10_0070 (Fig. 4C Topology match), whereas the disordered profiles of proteins *T. gondii* TGME49_091150 and *P. falciparum* PFL0360c poorly match (Fig. 4C Minimal match).

### Experimental validation of selected novel drCDC-UNK proteins

To explore the nature of drCDC-UNK proteins further, we selected a representative set of genes for epitope tagging by genetic knock-in in *T. gondii*. The selection criteria was designed to include examples of 1) genes expressed from moderate to abundant mRNA levels (62–100 percentile), 2) genes with a range of cyclical amplitude representing different dynamic profiles, 3) genes with distributed timing of peak expression representing all cell cycle phases of *T. gondii* tachyzoite division, and finally 4) genes encoding proteins of different predicted mass from 140 to 4,000 amino acids (aa). A total of 21 drCDC-UNK genes were selected (Table 1) along with one drCDC-UNK positive control (ISP1, gene ID TGME49_060820, for VSL2B graph see Fig. S2) [29]. Protein tagging in the gene locus was accomplished through the introduction of a triple copy of the hemaglutinin (HA) epitope into the predicted C-terminus in the *T. gondii* RH*Δku80* strain [30], [31] and individual protein expression and localization was determined by immunofluorescence assay (IFA) using suitable co-markers.

**Table 1:** drCDC-UNK genes selected for epitope tagging by gene knock-in in *T. gondii* tachyzoites.

*T. gondii* (Tg) gene ID TGME49_	Tg gene ID TGGT1_	Tg protein size (aa)	Tg mRNA percentile	Tg peak mRNA	Tg Localization^a^	*P. falciparum* (Pf) gene ID	Pf protein size (aa)	Pf mRNA percentile	Pf peak mRNA
028490	084050	344	90	G1	nuclear	PF13_0136	376	82	G1
030160	117730	142	100	G1	membrane	PF13_0058	143	100	S/M
119730	21560	149	87	G1	nd	PFB0620w	154	82	G1
005740	063160	1306	72	G1/S	ER	MAL8P1.105	1133	87	G1
025320	080520	504	92	S	dense granule	PF11_0373	679	93	S/M
040380	050490	4955	79	S	nd	PF13_0079	2029	75	S/M
060500	009700	270	98	S	nd	PF14_0092	262	99	S/M
060820	009340	176	96	S	ISP1	PF10_0107	144	66	S/M
094790	075110	351	99	S	microneme	PFD0955w	325	92	S/M
111880	088130	728	91	S	cytosol	MAL13P1.152	861	88	S/M
005320	005320	655	80	S/M	basal complex, internal memb.	MAL7P1.125	880	78	S/M
009200	022240	815	75	S/M	nd	PFL1025c	711	64	S/M
030350	117630	1254	81	S/M	rhoptry neck	PF14_0607	1071	98	S/M
033810	113520	2251	75	S/M	cytosol	PFB0190c	2295	98	S/M
035130	070970	487	75	S/M	centrosome, apical cap	PFB0475c	446	97	S/M
036510	069340	843	66	S/M	mitochondria	PF11_0467	629	62	S/M
041000	049670	233	93	S/M	rhoptry bulb	PF14_0572	193	86	S/M
052430	001330	767	64	S/M	membrane	PFI0540w	1165	97	S/M
064990	058220	259	98	S/M	membrane	PF14_0333	359	69	S/M
089100	034150	698	80	M/C	microneme	PFI0175w	742	95	S/M
112630	088920	2705	93	C/G1	nd	MAL13P1.308	2605	99	S/M
113860	090380	2105	62	C/G1	cytosol	PFD0900w	2011	91	S/M

**Figure 5:**
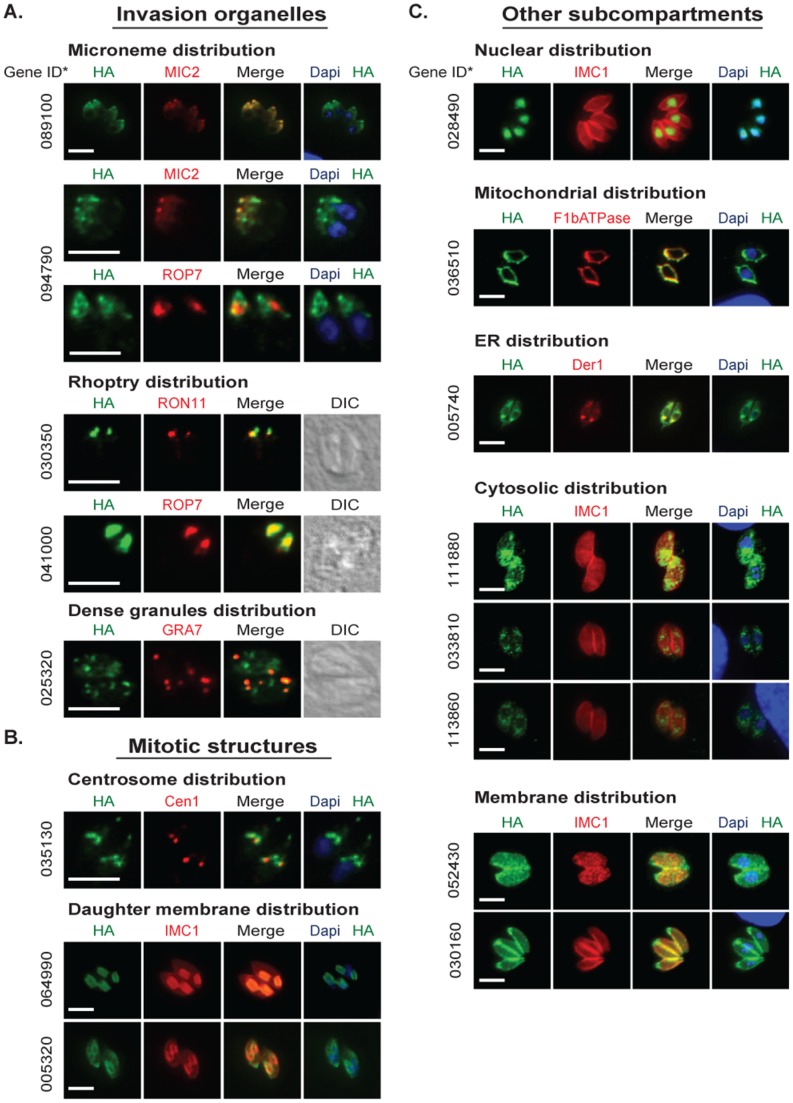
*T. gondii* drCDC-UNK proteins are enriched for invasion and mitotic proteins. Selected drCDC-UNK *T. gondii* genes representing a range of expression, protein size, and cell cycle timing were epitope tagged (HA = green) by genetic knock-in (see Material and Methods). Immunofluorescence analysis was performed on cloned transgenic strains to determine cellular localization of each drCDC-UNK protein. Representative images displayed here identified a variety of localizations. In all the image panels the drCDC-UNK protein tagged is shown in green. [**A**.] Proteins localized to invasion organelles were detected with co-markers (red) for: microneme (MIC2), rhoptry neck (RON11), rhoptry bulb (ROP7), dense granules (GRA7), and nuclear DNA stain = DAPI (blue). Direct interference contrast (DIC) images were included for the rhoptry and dense granule proteins. [**B**.] Proteins localized to mitotic structures were stained with co-markers (red) for staining the centrosome (centrin1 = Cen1), inner membrane complex (IMC1) and DAPI (blue) for the nucleus [**C**.] Proteins in other sub compartments were stained with co-markers: DAPI (blue) for the nucleus, co-markers (red) for mitochondria (F1**β** ATPase), ER (Der1), and membrane (IMC1). The drCDC-UNK gene IDs correspond to ToxoDB (http://www.toxodb.org/toxo/) assignment for the Type II ME49 strain and are indicated to the left of each image series omitting the common “TGME49_” pre-label. Scale bar  = 6 µm for each panel.

mRNA expression percentiles were obtained from EuPathDB, peak cell cycle times were obtained from Behnke, et al. (2010) Plos One and Bozdech, et al. (2003) Plos Biology

a -Localization in TGGT1_ parasites as shown in Fig. 5

nd = not determined.

Epitope tagging was successful for 16 of 21 genes (75%) as determined by IFA (Fig. 5) and Western analysis (Fig. S9), which is similar to the published success rate in the RH*Δku80* strain [30]. A variety of localization patterns were observed with particular enrichment (5/16 = 31%) for proteins showing apical-specific distribution (Fig. 5). When combined with proteins found to be associated with budding the majority of the proteins tagged were localized to specialized structures of apicomplexan division and invasion (8/16 = 50%, see Fig. 5 left panel and Table 1). Many of the mRNAs encoding these proteins reach peak expression in the S/M phase of the *T. gondii* tachyzoite cell cycle (Table 1 and Fig. S11), which is consistent with the timing of mRNAs encoding invasion and structural proteins in these parasites [10]. In general, Western analysis of the epitope tagged protein confirms the predicted protein mass (Fig. S9) with a few exceptions (see Fig. S10). Thus, these results confirmed our original prediction that cell cycle timing in the S/M/C periods would be a valuable characteristic for identifying novel proteins involved in apicomplexan replication.

The different fluorescent patterns of tagged drCDC-UNK proteins labeled many of the known sub compartments and novel structures within the *T. gondii* tachyzoite with proteins concentrated in the very apical end, the apical cap, the region between the cap and nucleus, around the nucleus, in the nucleus and contained in membranes. Five drCDC-UNK proteins were localized to invasion organelles including the apically located micronemes and rhoptries as well as dense granules. The proteins encoded by genes TGME49_089100 and TGME49_094790 were co-localized with MIC2 indicating these proteins are new microneme factors (Fig. 5A, top two left panels). TGME49_094790 also appears to partially co-localize with rhoptry bulb protein (ROP7) in the area posterior to the MIC2 co-localization. Additional independent experimentation will be required to validate that this factor is dual localized to microneme and rhoptry organelles. The mRNAs encoding these two putative microneme proteins displayed a cyclical profile with a peak in mitosis and cytokinesis that is characteristic of many other microneme mRNAs (Fig. S11) [10], [32]. However, neither of these newly identified microneme proteins are predicted to have a signal peptide suggesting that they are not likely to be secreted and are more likely to be escorter proteins similar to MIC7, which also lacks a signal peptide [33]. The TGME49_089100 protein is predicted to have a Hook domain that is known to mediate attachment to microtubules and may aid in microneme translocation along the subpellicular microtubules from the Golgi to the apical end of the parasite [34]. TGME49_094790 partially co-localized with MIC2 and has an mRNA pattern that more closely matches the profile of MIC13 mRNA than MIC2 (Fig. S11). The recent finding demonstrating microneme proteins organize into distinct sub compartments may be reflected in these different mRNA patterns [35]. The proteins encoded by genes TGME49_089100 and TGME49_094790 are the 18^th^ and 19^th^ proteins identified in the microneme organelle and therefore named MIC18 and MIC19 respectively. Two other drCDC-UNK proteins had distinctive concentrations that extended to the apical tip that was similar to rhoptry localization. The TGME49_030350 mRNA peaks in the S/M phase similar to other known rhoptry mRNAs (Fig. S11) [10] and the coding sequence is predicted to encompass an EF-hand domain and include a high number of strain-specific non-synonymous SNPs (n = 47), which are also features shared with other rhoptry proteins [36], [37]. During this study the TGME49_030350 gene was independently confirmed to be a new rhoptry neck protein, now designated RON11 and using anti-RON11 antibodies we validated that assignment by IFA analysis [38]. The protein encoded by the gene TGME49_041000 also displayed rhoptry localization. The co-localization of this factor with rhoptry bulb protein, ROP7, indicated that it is likely a novel rhoptry bulb protein. Like RON11 above, the mRNA encoding TGME49_041000 peaks in the S/M phase (Fig. S11). Interestingly, TGME49_041000 has 4 transmembrane domains similar to TgDHHC7 (Asp-His-His-Cys) that localizes to the rhoptry and affects apical positioning of rhoptries [38], [39]. TGME49_041000 is the 51^st^ protein identified in the rhoptry organelle and is therefore named ROP51. The fifth and last invasion organelle protein uncovered here is gene TGME49_025320, which encodes a protein with peak expression in S phase that partially localizes with the dense granule protein GRA7 (Fig. 5A). This gene has a potential signal peptide (D-score >0.5) and only has orthologs within Apicomplexa indicating it is a phylum specific invention consistent with the putative dense granule assignment. Unlike most dense granule mRNAs [10], the TGME49_025320 mRNA pattern is dynamic and cyclical (Fig. S11) indicating this factor is cell cycle regulated. The partial co-localization with GRA7 suggests this protein may have functions that are independent of this organelle or that this protein does not tolerate C-terminal tagging and is mistargeted to some dense granules and other vesicles by default.

Three proteins tagged in this study localized to specialized mitotic structures (Fig. 5B). Centrosome proteins show localization at the apical side of the nucleus in a structure that duplicates at the initiation of budding (Suvorova and White, unpublished and [40]). The centrosome is composed of internal centriole cores and surrounding peri-centriolar matrix (PCM) that is the assembly site of a specialized fiber that mediates the connection of the centrosome to the developing daughter buds [41]. Protein TGME49_035130 is encoded by a S/M peak mRNA similar to inner membrane complex sub-compartment protein 1 (ISP1) and Sas-6-like (SAS6L) mRNAs (Fig S5) and displayed PCM as well as apical cap localization. This protein surrounds centrin1 (Cen1), which is a marker of the distal end of the centriole (Fig. 5B). TGME49_035130 is conserved only in apicomplexan parasites, which would be consistent with the low conservation of PCM proteins in other eukaryotes [42], [43]. This protein also co-localizes with apical protein ISP1 (data not shown). TGME49_064990 protein preferentially targeted to the daughter inner membrane complex (IMC) during parasite budding similar to IMC3 [44]. This protein had a tight cell cycle profile with peak expression in S/M (Fig. S11), and similar to other IMC membrane proteins, TGME49_064990 is predicted to be palmitoylated. Gene TGME49_005320 encodes the last factor we observed in parasite mitotic structures. This factor localized to the basal complex and IMC structures. This protein showed peak expression in the S/M phase of the cell cycle (Fig. S11).

The next group of proteins we tagged included eight drCDC-UNK proteins that displayed distinct localization to conventional eukaryotic cell compartments (see Fig 5C). This group included two cell cycle factors with potential functions across eukaryotic cells. A highly conserved novel nuclear protein encoded by gene TGME49_028490 had peak expression at G1 and is predicted to be phosphorylated (ascore = 100). Co-localization of this protein with DAPI (Fig. 5C) and antibodies against the *T. gondii* proliferating cell nuclear antigen (PCNA) protein (data not shown) confirmed it was exclusively located in the parasite nucleus. The gene, TGME49_036510, encoded a mitochondrial protein with a cyclical profile, which was confirmed by co-localization with the mitochondrial protein, F1**β** ATPase. In *P. falciparum*, synthesis of mitochondrial proteins occurs when the mitochondria mature during the S/M phase (Table 1) of the cell cycle [9] and the relative cell cycle timing likely applies to the *T. gondii* tachyzoite [10]. The TGME49_036510 protein contains a predicted GAF-like domain present in a wide variety of proteins including cGMP phosphodiesterases that are important regulators of signal transduction and are potential therapeutic targets. TGME49_005740 encodes for a protein that displays putative endoplasmic reticulum localization [32] due to its localization with DER1 and encodes for a highly conserved protein that contains a C-terminal transmembrane domain. The last three proteins of this group had extensive cytosolic distributions. The TGME49_111880 mRNA displays peak expression in S phase (Table 1), and the encoded protein is predicted to be phosphorylated. The protein encoded by gene TGME49_033810 displays punctate cytosolic localization consistent with vesicles and displays peak expression at S/M (Table 1). The TGME49_033810 protein contains four predicted Sel1 domains that have been implicated in negative regulation of the notch developmental pathway in *C. elegans*. We identified a protein with peak expression at C/G1 phases (Table 1) encoded by TGME49_113860, which suprisingly localized to the cytosol. The G1 image pattern for this factor was selected for comparisons with the two other cytosolic factors we discovered, however, this factor was found to increase dramatically in the newly formed daughter cytosol consistent with the profile of the encoded mRNA (data not shown). TGME49_113860 contains a predicted regulator of chromosome condensation (RCC1) repeat, suggesting this protein may have an interesting role in cell division.

The final group of two proteins (Fig. 5C, bottom two panels) was found to associate with the plasmalemma or inner membrane structures. Protein TGME49_052430 was concentrated in the parasite plasmalemma extending beyond the IMC and into the apical tip of the parasite. This protein contains a predicted Bet v1-like domain that has been implicated in lipid binding and lipid transporter activity and peaks in S/M similar to known membrane proteins: IMC1 and Sag Related Sequence 12B (SRS12B) (Fig. S11).

TGME49_030160 encodes an interesting membrane protein that stains the complete parasite with a concentration in the apical end of the parasite and is absent in the developing daughters during endodyogeny. This protein shows peak expression in G1 (Fig. S11) and contains a coil-coil domain that may aid in its membrane localization. Independently from our studies, this protein was localized to the membrane after overexpression and fusion to GFP [45] corroborating our findings. The localization of TGME49_030160 resembles another *T. gondii* protein, TgPhIL1 [46] that is thought to be required to help tether the inner membrane complex to the plasma membrane however, despite the similar localization pattern the mRNA patterns do not match (data not shown) and the mRNA pattern for TGME49_030160 more closely resembles IMC12 and IMC13 (Fig. S11), which are known to peak in G1 as well.

### Building additional clues to function

**Figure 6:**
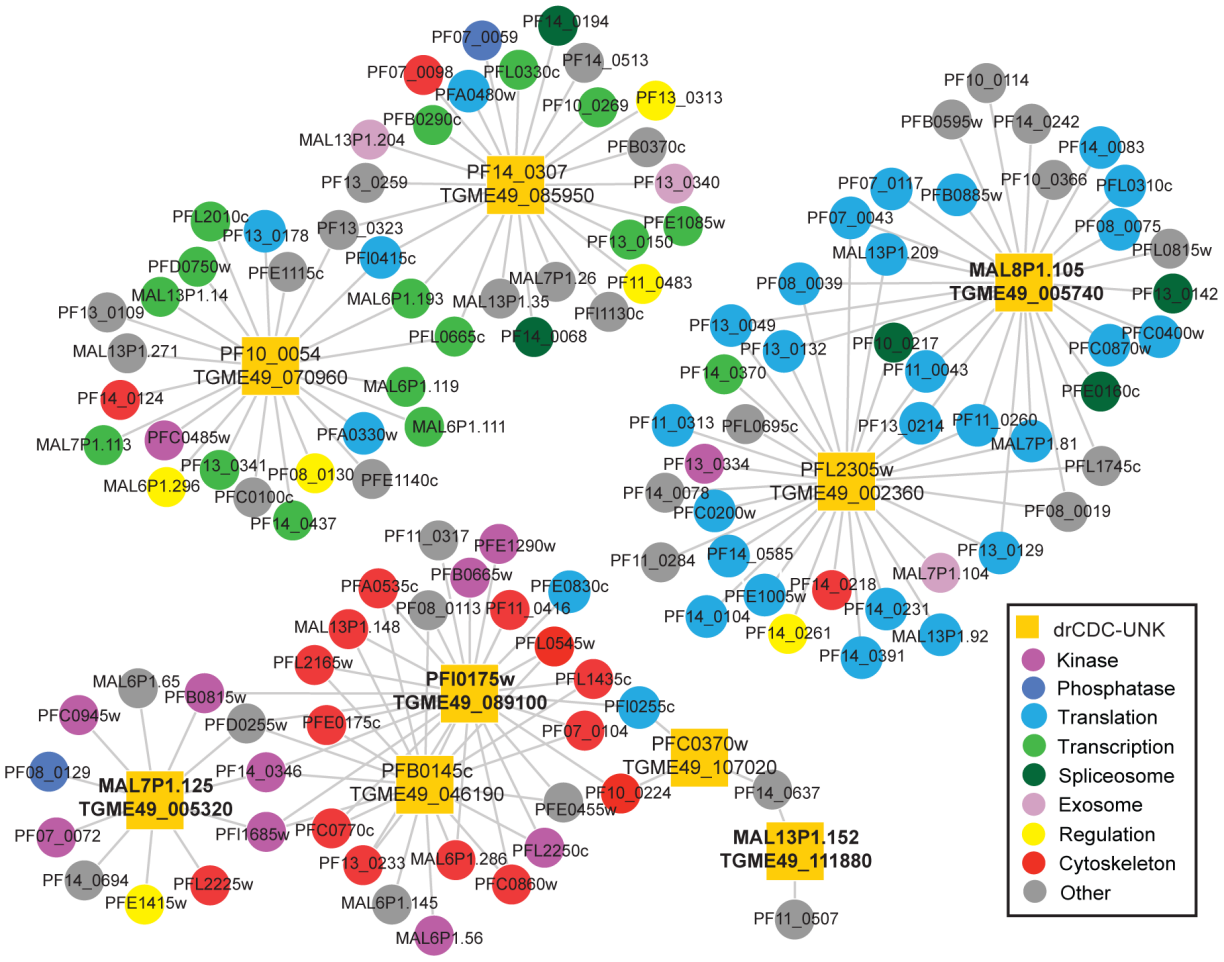
*T. gondii* and *P. falciparum* network analysis of drCDC-UNK proteins. From the 125 orthologous drCDC-UNK *P. falciparum* genes, 9 proteins (yellow squares) were identified in the yeast two-hybrid analysis to be involved in 3 sub networks. Specifically, we found 6 orthologs interacted with cytoskeletal components and cell cycle kinases (bottom left), while 2 ortholog pairs were linked to transcription factors (top left) or protein synthesis factors (top right). Where the corresponding *T. gondii* ortholog was analyzed by IFA (Fig. 5) the *T. gondii* gene ID is indicated by bold font.

Biological interaction networks can be inferred using conserved protein interactions or interlogs [47], [48], and while there is no global interactome data reported in *T. gondii*, there is a partial high-throughput yeast-two hybrid interactome in *P. falciparum*. We used this *P. falciparum* network that represents ∼25% of the total proteome [49] and includes a compilation of 2,849 interactions between 1,304 proteins [49] measured with an adapted yeast two-hybrid method to construct a *P. falciparum* interactome encompassing nine of the drCDC-UNK proteins (see Dataset S2). This analysis revealed several interactions (Fig. 6 and Dataset S2) that extend the information obtained by protein localization in *T. gondii* (Fig, 5).

A large sub network (Fig. 6) comprising five of the drCDC *P. falciparum* proteins showed linkage to major groups of kinases (9) and cytoskeletal proteins (14). For three of the drCDC *P. falciparum* proteins the corresponding *T. gondii* orthologs were epitope tagged (Fig. 5 tagged proteins TGME49_005320, TGME49_089100, and TGME49_111880) and found to localize to the basal complex/internal membrane, micronemes, and cytosol, respectively, lending key biological information to these potential interactions. The MAL7P1.125/TGME49_005320 ortholog pair is predicted to interact with five kinases including two calcium-dependent and two cyclic nucleotide kinases (Dataset S2) that are thought to work closely together to control motility, invasion and egress [50], [51] in *P. falciparum*. This information together with the localization of the *T. gondii* ortholog to the basal complex/internal membrane of the tachyzoite suggests a role for this factor in building the parasite invasion apparatus, and adds to the interests in these kinases as possible drug targets [52]. Protein PFI0175w (paired with TGME49_089100) was linked to several cytoskeletal proteins and the *T. gondii* protein ortholog localized to the micronemes (Fig. 5A). As noted earlier, TGME49_089100 has the potential to interact with microtubules through a hook domain that is also conserved in the *P. falciparum* ortholog. Another interaction of interest within the large sub network is the potential of PFB0145c (paired with TGME49_046190) to interact with the cytoskeletal components. The predicted prefoldin domain in this factor is known to aid in the assembly of actin [53]. Also consistent with a role in invasion, is a piggyBac insertion mutant that disrupts *P. falciparum* PFB0145c leading to a severe growth defect in the intra-erythrocytic cycle of the merozoite [54].

Two other smaller sub networks (Fig. 6 top right and left) were identified by this analysis. The first involves a *T. gondii* protein encoded by TGME49_005740 for the corresponding drCDC *P. falciparum* ortholog (MAL8P1.105) that was found localized to the parasite endoplasmic reticulum (Fig. 5C). It may be no coincidence that this is a major site of ribosome localization in cells and the network interactions shown here involve twenty-five translational factors. Finally, the last protein subnetwork (Fig. 6 top left) includes many transcription related factors and this could be related to the RNA methyltransferase domain in PF14_0307/TGME49_085950 and the possible nucleic acid binding site in the C-terminal end of the PF10_0054/TGME49_070960 protein pair.

## Discussion

Eukaryotic cell cycles are preserved in spatial and timing relationships [55] that are independent of chromosome evolution. Lineages that are represented by *P. falciparum* and *T. gondii* are thought to have diverged several hundred million years ago [3] and, as a consequence, their chromosome structures have become very distinct. The nucleotide bias is substantially different and synteny from the apicomplexan common ancestor has been completely lost [23]. Despite this divergence, evidence presented here builds on discoveries of a dominant selection of gene expression timing [9], [10] preserved to carryout the unique topology of daughter parasite assembly and replication. Distinct sub-transcriptomes in the Apicomplexa deliver proteins in a “just-in time” assembly [9], [10] and they also separate into the two halves of the cell cycle many apicomplexan specific inventions, such as building invasion organelles and internal daughter structures (S/M peak) from more ancient eukaryotic functions such as constructing transcription/translation and DNA replication machineries (G1 peak) [10].

In 2002 we entered the genome era for Apicomplexa research with the complete sequencing of the *P. falciparum* genome [8], [56], [57] however, almost half of the genes discovered in these parasites have yet to be characterized and are still classified as hypothetical or unknown. Many of these hypothetical genes could be essential for parasite growth and offer unique targets for therapeutic development if we understood their functions. Large-scale studies of hypotheticals have been notoriously difficult to accomplish, and thus far, only one effort has launched a genome-wide gene knockout effort in the Apicomplexa that could capture the function of some of these genes [54]. Short of expanding these high-through-put genetic approaches, the sorting strategies based on co-expression used here have benefits in discovering important Apicomplexa proteins involved in building new parasites. The strategy devised was an easily implemented decision tree analysis to identify novel proteins through relaxed orthology criteria, conserved mRNA peak timing and novel protein topology. The modified criteria for orthology assignment identified nearly 600 additional protein pairs than found using conventional methods that do not adequately account for additions of intrinsically disordered tails or loops that lead to penalized scores in conventional algorithms. Using cell cycle expression data to sort genes, we resolved genes that are uniquely cell cycle regulated in each parasite from those that form a core set of cycling mRNAs likely conserved across the parasite family. Thus in *P. falciparum*, genes encoding cell cycle regulated surface and unique export proteins sorted into a CDC list that lacked conservation in *T. gondii*, while rhoptry proteins found in *T. gondii* and other coccidians fell into an opposite class. The CDC genes specific to each parasite include a large number of novel proteins representing undiscovered molecular features necessary for completing the *T. gondii* and *P. falciparum* life cycles in each unique host cell environment. These genes were not studied here, but are as worthy of serious investigation as the dual cell cycle regulated genes that were the focus of this study.

The ∼700 genes (9% in *T. gondii* and 12.8% HB3 *P. falciparum*) that were found to be dual cell cycle regulated and conserved in *T. gondii* and *P. falciparum* represent many of the core set of CDC genes in apicomplexan parasites. This is a similar number of core CDC genes conserved in fungal and mammalian eukaryotic models (5–7%) [58] and the canonical CDC genes present in our lists overlaps these previous reports. Importantly, included in this set of core CDC genes are more than a hundred genes (drCDC-UNK genes) classified as hypothetical in *T. gondii* and *P. falciparum*. As a group these proteins have higher proportion of disordered domains and in *T. gondii* these proteins tended to be longer. Given the lack of functional insight it is easy to assume these proteins are weakly conserved, perhaps even false positive cases of orthology assignment. However, this study demonstrates these proteins have remarkable conserved topology and their expression at the mRNA level is well preserved in cell cycle timing. The drCDC-UNK genes are also conserved in the levels of encoded mRNA abundance with the exception of the group of hypothetical CDC genes in *P. falciparum* that had peak expression during late nuclear reduplication and parasite budding (S/M timing, Fig. S2). Here the uniform high abundance of expression of these novel CDC genes set them apart from their *T. gondii* counterparts. We believe this indicates a dominant role for these proteins in organizing and building daughter parasites that have a 10-fold scaling difference between the replication of the merozoite of *P. falciparum* compared to the tachyzoite of *T. gondii*. This theme again was borne out in the lack of regulatory proteins present in the hypothetical group of core CDC genes and the tagging of 16 of these genes in *T. gondii* that revealed newly discovered proteins of invasion organelles and membrane structures, which are all scalable building components of the infectious parasite. The results of tagging hypothetical proteins also highlights the amount of biology we still must uncover in these parasites.

Our results clearly demonstrated that screening hypothetical genes by mRNA timing, orthology and protein topology enhances our ability to find specific types of proteins such as novel invasion proteins based on co-expression. The next steps in uncovering the function of the 125 drCDC-UNK proteins will be to use available genetic models to define phenotypes and interactor studies to identify protein partners, which is beyond the scope of this initial study. Currently, there is little global interactome data available and more interactome data is needed. As more interactome data becomes available for the Apicomplexa phylum it will be increasingly easier to predict protein function and help prioritize large lists of hypothetical proteins based on predicted functions for genome wide knockout strategies.

## Materials and Methods

### Cell culture

Parasites were maintained as previously described [59] in primary human foreskin fibroblasts (HFF) kindly provided by Dr. David Roos. The parasite strains RHΔ*ku80* and RH*ΔhxgprtΔku80* were used for endogenous tagging of genes as previously described [30]. Stable transgenic parasite lines were selected in media containing 1 µM pyrimethamine.

### Sequence analysis and ortholog identification

Gene/protein sequences of the ME49 and GT1 strains of *T. gondii* were obtained from www.toxodb.org, toxoDB V7.1, while gene/protein sequences of the HB3 strain of *P. falciparum* were retrieved from www.plasmodb.org, V8.0. The predicted models for *Toxoplasma* and *Plasmodium spp*. genes are still being refined and therefore, it is possible there are differences between predicted protein size and the native proteins due to artifacts of incorrect calling of the translation start/stop sites or introns. While imperfect, we used the current annotated protein sequences to analyze for the presence of known domains, motifs and repeats using SMART (http://smart.embl-heidelberg.de). Gene expression data using spline curves, as previously described [10] for *T. gondii* and gene expression data from the DeRisi lab available on www.plasmodb.org [9] for the intraerythrocytic cycle of *P. falciparum* were used to determine mRNA expression and timing. To determine orthologous pairs of proteins in *T. gondii* and *P. falciparum* we utilized all-versus-all BLASTP searches using the InParanoid script [60]. In particular, we accounted for all sequence alignments irrespective of any constraints of alignment length and score. Sequence pairs with mutually best scores were selected as central orthologous pairs. Homologous proteins of both species were clustered around these central pairs to form orthologous groups. The quality of such clusters was further assessed by a standard bootstrap procedure. Accounting for all pairs, we obtained a set of 2,781 orthologous protein pairs in *P. falciparum* and *T. gondii* (Dataset S1).

### *P. falciparum* mRNA half-life analysis

Cell cycle phases of peak mRNA abundance (expression data were obtained from [9]) and peak mRNA half-life [20] were manually compared for all 125 *P. falciparum* drCDC-UNK genes throughout the 48 h intra-erythrocytic life cycle. Where mRNA abundance and maximum half-life occurred in the same phase of the cell cycle the gene was scored a zero. An offset of one phase was scored a 1 when a gene's abundance peaked in a cell-cycle phase before the half-life reached its maximum (e.g. abundance peak in ring phase, half-life peaks in trophozoite phase). In turn, an offset of one phase was scored as -1 when a gene's abundance was maximal in a cell-cycle phase after the half-life reached its peak (e.g. abundance peaks in trophozoite phase, half-life peaks in ring phase). All mRNA abundance and half-life data was obtained from PlasmoDB.

### Protein intrinsic disorder predictions

Two principally different approaches were used to identify the intrinsic disorder propensities in proteins. To these ends, we applied binary classifiers that classify whole proteins as either mostly disordered or mostly ordered and a disorder predictor that provides per-residue disorder propensity for a query protein. The two binary predictors of intrinsic disorder used were charge-hydropathy plot (CH-plot) [61], [62] and cumulative distribution function analysis (CDF) [62]. These methods perform binary classification of whole proteins as either mostly disordered or mostly ordered [62].

We also utilized the combined CH-CDF analysis, where the coordinates of each spot are calculated as a distance of the corresponding protein in the CH-plot (charge-hydropathy plot) from the boundary (Y-coordinate) and an average distance of the respective cumulative distribution function (CDF) curve from the CDF boundary (X-coordinate) [63], [64]. The primary difference between CH and CDF binary predictors is that the CH-plot is a linear classifier that takes into account only two parameters of the particular sequence (charge and hydropathy), whereas CDF predictor was trained to distinguish order and disorder based on a significantly larger feature space. Therefore, CH-plot analysis is predisposed to discriminate proteins with substantial amount of extended disorder (random coils and pre-“molten globules”) from proteins with compact conformations (“molten globule”-like and rigid well-structured proteins). On the other hand, CDF analysis may discriminate all disordered conformations, including molten globules and mixed proteins containing both disordered and ordered regions, from rigid well-folded proteins. Thus, the CH-CDF analysis enables discrimination of proteins with extended disorder from potential molten globules and mixed proteins.

Positive and negative Y values in corresponding CH-CDF plot correspond to proteins predicted within CH-plot analysis to be intrinsically disordered and extended or compact, respectively. On the other hand, positive and negative X values are attributed to proteins predicted within the CDF analysis to be ordered or intrinsically disordered, respectively. Thus, the resultant quadrants of CDF-CH phase space correspond to the following expectations: Q1, proteins predicted to be disordered by CH-plots, but ordered by CDFs; Q2, ordered proteins; Q3, proteins predicted to be disordered by CDFs, but compact by CH-plots (i.e., putative molten globules or mixed proteins); Q4, proteins predicted to be disordered by both methods (i.e., proteins with extended disorder). This CH-CDF analysis was applied to the set of the 125 drCDC-UNK proteins in order to their overall order/disorder predispositions at the whole molecule level.

PONDR VSL2B plots were analyzed to evaluate the disorder content in the protein sequences on the per-residue level as previously described [28]. PONDR VSL2B-based order/disorder (>0.5 = disorder) scores per amino acid residue were used to create corresponding profiles and a best-fit method to align the *T. gondii* and *P. falciparum* proteins was applied to each ortholog pair.

### Endogenous epitope tagging of *T. gondii* proteins

All designs for endogenously tagging of individual proteins in the genetic locus employed predicted gene models that were verified by RNA-sequencing data in ToxoDB (www.toxodb.org). For endogenous tagging, the vector pLIC-HA3-DHFR was generously provided by Vern Carruthers [30]. Briefly, genomic fragments were amplified by PCR for each gene (for primers used see Dataset S3) using a design that fuses the C-terminal end of each coding region with a triple hemagglutinin tag (HA3) in the LIC-HA3-DHFR plasmid. A unique restriction site upstream of the predicted stop codon was identified to perform the linearization. pLIC-HA3-DHFR was linearized with PacI and subjected to T4 DNA polymerase (Novagen, LIC-qualified) for ligation independent cloning (LIC). Sequenced constructs were electroporated into either RH*Δku80* or RH*ΔhxgprtΔku80* parent strains using standard methods. Transgenic strains were selected in media containing pyrimethamine (1 µM final). Polyclonal isolates were screened to verify homologous recombination using a unique 5′-primer upstream of each genomic fragment used to tag the locus in combination with a common HA primer (see Dataset S3) to confirm loss of the wild type locus. To confirm endogenous tagging, western blotting was performed (Fig. S9) as previously described [65], [66] using rat anti-HA antibodies (Roche) at a dilution of 1∶500 and goat anti-rat IgG conjugated to HRP (Jackson ImmunoResearch Laboratories, Inc., West Grove, PA) at 1∶1000. For proteins that did not show a match in predicted protein size genomic DNA was harvested from purified parasites and analyzed by PCR to verify the correct gene locus was tagged. PCR products were amplified with the primers in Dataset S3 using Taq DNA polymerase (New England Biolabs) and were analyzed after gel electrophoresis on a 0.8% agarose gel stained with SYBR safe DNA gel stain (Invitrogen).

### Immunofluorescence assay and microscopy

Immunofluorescence assays were performed as previously described [10]. Briefly, infected HFF monolayers were fixed in 3.7% paraformaldehyde and permeabilized on 0.25% Triton TX-100. After blocking in 1% PBS-BSA for at least 30 min, primary antibodies were added for one hour at room temperature. Primary antibodies were prepared at the indicated dilutions: rat anti-HA (Roche) at 1∶500, rabbit anti-HA (Abcam) at 1∶1000, mouse anti-MIC2 (kindly provided by Dr. Jean Francois Dubremetz) at 1∶2000, rat anti-RON11, mouse anti-ROP7 and mouse anti-GRA7 (all kindly provided by Dr. Peter Bradley) at 1∶1000, rabbit anti-Cen1 [22] at 1∶400, IMC1 (kindly provided by Dr. Gary Ward) at 1∶1000, and rabbit anti-GFP (Torrey Pines Biolabs Inc.) at 1∶000 for mitochondrial plasmid (pF1**β** ATPase-GFP) and ER plasmid (pDer1-RFP) in blocking buffer. Alexa Fluor-conjugated secondary antibodies (Molecular Probes, Life Technologies) were used at 1∶1000 dilutions in blocking buffer with the addition of DAPI (at 0.5 µg/mL) staining during the last 5 minutes of secondary antibody (Molecular Probes, Life Technologies) incubation. The secondary antibody was removed and coverslips were washed 3X with 1X PBS pH 7.4 then mounted in Aquamount (Thermo Scientific). Image acquisition was performed on a Zeiss Axiovert microscope equipped with 100x objective and images collected with a digital camera (SPOT, Dynamic Instruments Inc.) and processed in Adobe Photoshop CS v4.0 using linear adjustment for all channels in an intragroup fashion.

## Supporting Information

Figure S1 **Correlation between peak mRNA abundance and peak mRNA half-life.** Half-life mRNAs measurements and cell cycle assignments were retrieved [20] and the cell cycle phases of peak mRNA abundance [9] and peak mRNA half-life [20] were compared for all 125 drCDC-UNK genes in *P. falciparum*. The *P. falciparum* drCDC-UNK mRNAs that peak in the same phase of the cell cycle as their maximum mRNA half-life were assigned a score of zero. An offset of one phase (e.g. abundance peaks in ring, half-life peaks in trophozoite) was scored as 1, while the reverse relationship was scored -1. For 89% of the *P. falciparum* drCDC-UNK genes the peak level and half-life fell within one phase of each other.
(TIF)

Figure S2 **Topology matches for drCDC-UNK proteins (1–15 out of 32 profiles).** Order/disorder plots (VSL2B scores, >0.5 = disordered) of *T. gondii* (blue) versus *P. falciparum* (red) proteins that display matching topology protein pairs. Topology profiles for the first fifteen (1–15 out of 32 profiles) drCDC-UNK protein pairs with matching topology are shown. Order/disorder curves for drCDC-UNK protein pairs were aligned by best-fit methods, independent of order/disorder, and ordered by increasing protein length. Note that the set of matching orthologs includes examples of proteins that are nearly fully ordered (<0.5 score) as well as those that are nearly completely disordered (>0.5).
(TIF)

Figure S3 **Topology matches for drCDC-UNK proteins (16–32 of 32 profiles).** Order/disorder plots (VSL2B scores, >0.5 = disordered) of *T. gondii* (blue) versus *P. falciparum* (red) proteins that display matching topology protein pairs. Topology profiles for the next seventeen (16–32 out of 32 profiles) drCDC-UNK protein pairs with matching topology are shown. Order/disorder curves for drCDC-UNK protein pairs were aligned by best-fit methods, independent of order/disorder, and ordered by increasing protein length. The x-axis for longer proteins was extended to better display the protein secondary structure. Note that the set of matching orthologs includes examples of proteins that are nearly fully ordered (<0.5 score) as well as those that are nearly completely disordered (>0.5).
(TIF)

Figure S4 **Topology additions of N-terminal domains for drCDC-UNK proteins.** Order/disorder plots (VSL2B scores, >0.5 = disordered) of *T. gondii* (blue) versus *P. falciparum* (red) proteins that display additions of loops or tails for the protein pairs. Order/disorder curves for drCDC-UNK protein pairs were aligned by best-fit methods, independent of order/disorder, and ordered by increasing protein length. The x-axis for longer proteins was extended to better display the protein secondary structure. Topology profiles of drCDC-UNK proteins with N-terminal domain additions in either species in most cases the domain addition was highly disordered.
(TIF)

Figure S5 **Topology additions of C-terminal tails for drCDC-UNK proteins.** Order/disorder plots (VSL2B scores, >0.5 = disordered) of *T. gondii* (blue) versus *P. falciparum* (red) proteins that display additions of loops or tails for the protein pairs. Order/disorder curves for drCDC-UNK protein pairs were aligned by best-fit methods, independent of order/disorder, and ordered by increasing protein length. The x-axis for longer proteins was extended to better display the protein secondary structure. Unique C-terminal additions were observed for 15 drCDC-UNK pairs. Similar to N-terminal additions, the C-terminal extension was species specific and disordered.
(TIF)

Figure S6 **Topology additions of complex additions of tails and loops in drCDC-UNK proteins (1–21 of 41 profiles).** Order/disorder plots (VSL2B scores, >0.5 = disordered) of *T. gondii* (blue) versus *P. falciparum* (red) proteins that display complex additions were observed in 41 drCDC-UNK pairs and included combinations of N and C-terminal tails or loops. The first 21 profiles of complex additions are shown here. Order/disorder curves for drCDC-UNK protein pairs were aligned by best-fit methods, independent of order/disorder, and ordered by increasing protein length.
(TIF)

Figure S7 **Topology additions of complex additions of tails and loops in drCDC-UNK proteins (22–41 of 41 profiles).** Order/disorder plots (VSL2B scores, >0.5 = disordered) of *T. gondii* (blue) versus *P. falciparum* (red) proteins that display additions of loops or tails for complex additions were observed in 41 drCDC-UNK pairs and included combinations of N and C-terminal tails or loops. The next 20 profiles of complex additions are shown here. Order/disorder curves for drCDC-UNK protein pairs were aligned by best-fit methods, independent of order/disorder, and ordered by increasing protein length. The x-axis for longer proteins was extended to better display the protein secondary structure.
(TIF)

Figure S8 **Minimal topology matches for drCDC-UNK proteins.** Order/disorder plots (VSL2B scores, >0.5 = disordered) of 15 *T. gondii* (blue) versus *P. falciparum* (red) proteins that display minimal topology matched protein pairs. Order/disorder curves for drCDC-UNK protein pairs were aligned by best-fit methods, independent of order/disorder, and ordered by increasing protein length. The x-axis for longer proteins was extended to better display the protein secondary structure.
(TIF)

Figure S9 **Western analysis of epitope tagged** ***T. gondii*** **drCDC-UNK proteins.** Western blot analysis of protein extracts from *T. gondii* drCDC-UNK tagged strains in Fig. 5 were detected by anti-HA antibody to verify endogenous tagging. Gene IDs correspond to ToxoDB (http://www.toxodb.org/toxo/) assignment for the Type II ME49 strain and are indicated above each western blot image omitting the common “TGME49_” pre-label. The protein marker to the left of each western blot is shown in kDa. Predicted protein sizes from ToxoDB are shown below each gene ID in parenthesis and include the additional 4.4 kDa for the triple HA tag. Asterisks indicate the full-length protein detected by western blot and arrows indicate possible cleaved or degradation products. The analysis showed that 12 out of 16 HA-tagged proteins migrated on Western blots with a mass close to the annotated protein size. Three predicted membrane proteins (TGME49_005320, TGME49_052430 and TGME49_030160) showed anomalous protein sizes, while the protein encoded by TGME49_041000, could not be detected by western analysis. PCR analysis to verify the correct gene knock-in of the 3xHA tag into the appropriate gene locus for these four exceptions are shown in Fig. S10.
(TIF)

Figure S10 **PCR analysis of 3xHA knock-in into the genetic locus of TGME49_041000, TGME49_005320, TGME49_052430 and TGME49_030160.** PCR analysis was used to verify tagging of the gene at the correct locus using primers from Dataset S3 for the four drCDC-UNK proteins that failed western blot analysis (Fig. S9). For each of the four PCRs lane 1 shows the genomic DNA (gDNA) from the corresponding endogenously tagged strain amplified with a forward verification primer and a common reverse primer specific for the 3xHA cassette insertion. Lane 2 shows the PCR results from parent strain (RH) gDNA with the forward verification primer and the common 3xHA reverse primer (negative control). Lane 3 shows the PCR result confirming that parent strain gDNA contains the native gene locus; the forward verification primer was combined with a reverse primer binding to native gene sequence that is downstream of the gene knock-in site (present only in the parent gDNA). M = marker and the size of each PCR product is shown above the corresponding lane in base pairs (bp). The analysis demonstrates that all four genes were tagged at the correct locus as evidence by the correct size PCR product for each tagged strain that is larger than the native DNA fragment produced from the native locus present in the parent gDNA.
(TIF)

Figure S11 **Co-expression graphs for selected drCDC-UNK mRNAs.** The mRNA expression [10] for ten *T. gondii* drCDC-UNK genes (blue line) endogenously tagged in Fig. 5 was compared to previously characterized *T. gondii* genes (green and red lines) that show a conserved timing of mRNA expression among genes that code for proteins with similar functions. See Behnke et al. 2010 and ToxoDB for cell cycle data for all genes listed and gene IDs for the characterized genes (green and red lines) used to show conserved timing of mRNA peak expression. Plots of mRNA expression profiles show mRNA expression levels (RMA value, y-axis) throughout the 8.75 h synchronized lifecycle of *T. gondii* tachyzoites (in hours, x-axis). Due to the method of synchrony that arrests *T. gondii* RH^TK+^ tachyzoites in late G1/early-S transition (thymidine block and release) [4], [10], we partitioned the cell cycle into early G1 = 4.6–6.5 h, late G1 = 6.6–8.75 h, and S/M/C = 0–4.5 h.
(TIF)

Dataset S1 **Decision tree analysis datasets used to identify conserved Apicomplexa CDC genes.** All datasets used in the decision tree analysis described in Fig. 1A to identify the 744 drCDC genes and the 125 drCDC-UNK genes. Gene annotations and the peak mRNA expression timing are included for all genes.
(XLSX)

Dataset S2 ***T. gondii*** **and** ***P. falciparum*** **network analysis dataset of drCDC-UNK proteins and their potential protein interactions.** A list of proteins and their predicted interactions from the network analysis of drCDC-UNK proteins is shown in Fig. 6.
(XLSX)

Dataset S3 **Primers for endogenous tagging T. gondii drCDC-UNK genes.** Primers designed to endogenously tag the drCDC-UNK genes listed in Table 1 and to verify endogenous tagging at the correct locus (Fig. S10).
(XLSX)
