# Supplementary figures and images for: Identifying Novel Cell Cycle Proteins in Apicomplexa Parasites through Co-Expression Decision Analysis
Source: PLoS One. 2014 May 19;9(5):e97625. doi: 10.1371/journal.pone.0097625 (PMC4026381; doi:10.1371/journal.pone.0097625)

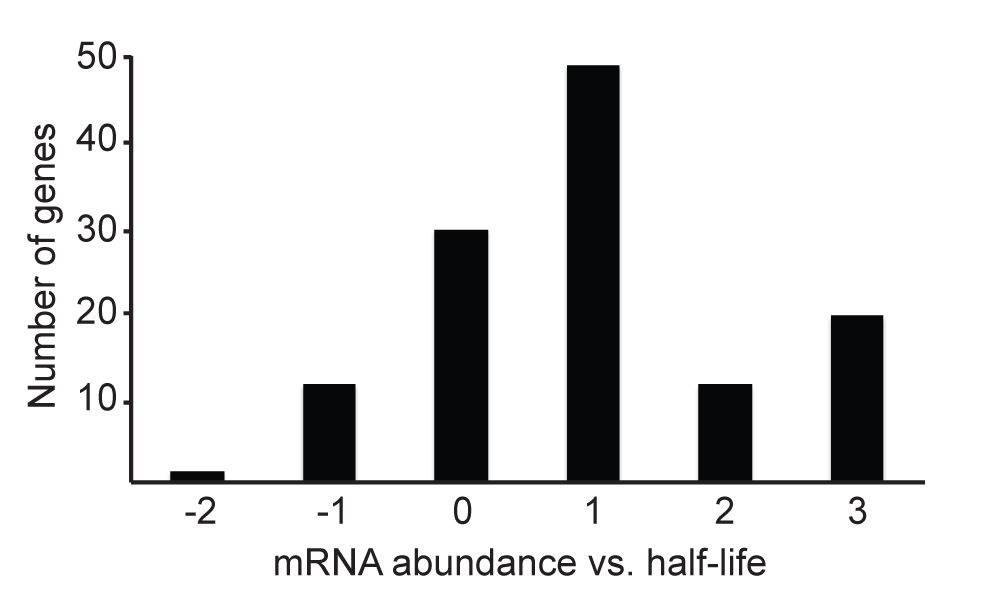

Supplement: Figure S1 — Correlation between peak mRNA abundance and peak mRNA half-life. Half-life mRNAs measurements and cell cycle assignments were retrieved [20] and the cell cycle phases of peak mRNA abundance [9] and peak mRNA half-life [20] were compared for all 125 drCDC-UNK genes in P. falciparum. The P. falciparum drCDC-UNK mRNAs that peak in the same phase of the cell cycle as their maximum mRNA half-life were assigned a score of zero. An offset of one phase (e.g. abundance peaks in ring, half-life peaks in trophozoite) was scored as 1, while the reverse relationship was scored -1. For 89% of the P. falciparum drCDC-UNK genes the peak level and half-life fell within one phase of each other. (TIF) [file pone.0097625.s001.tif]

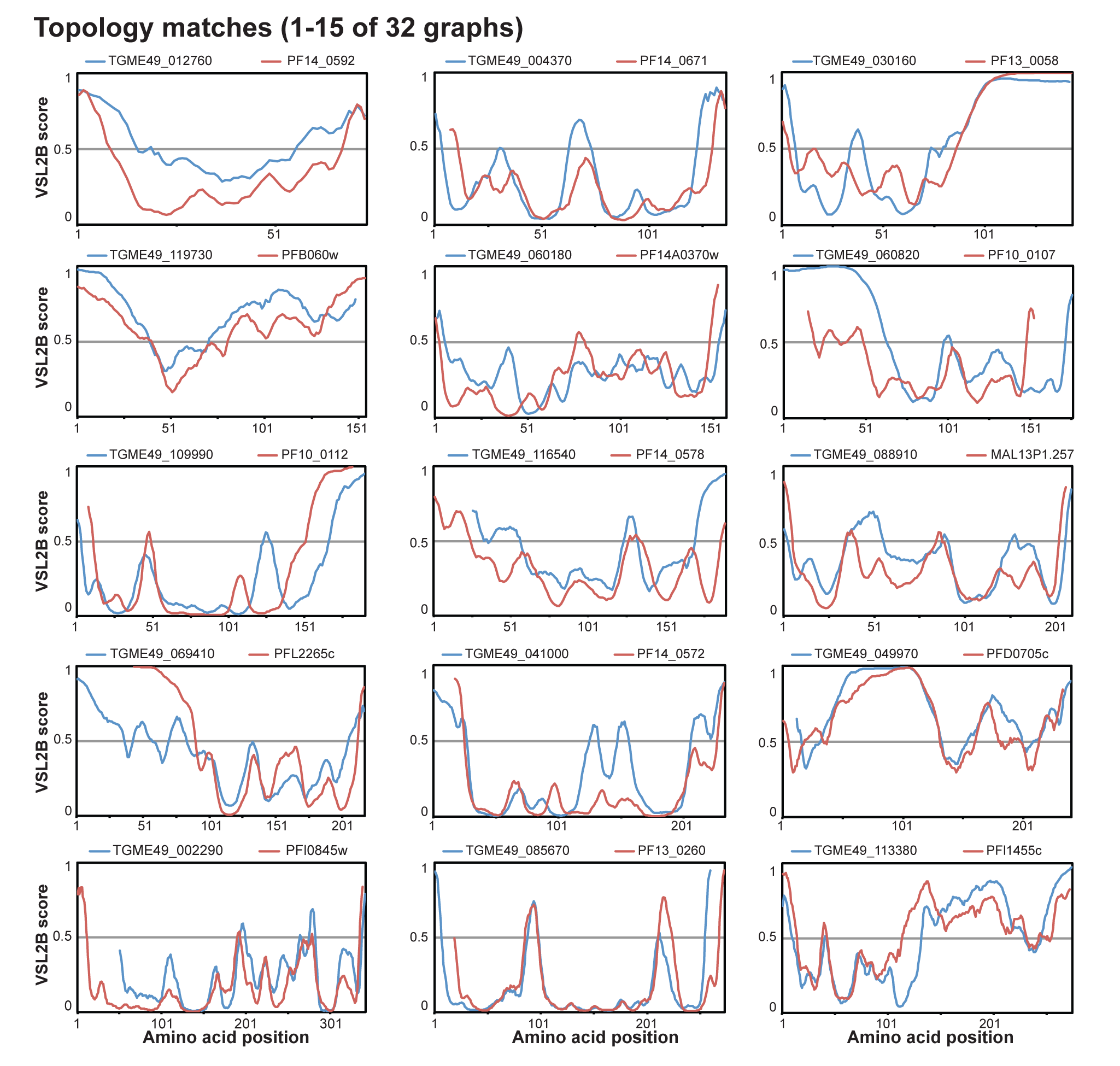

Supplement: Figure S2 — Topology matches for drCDC-UNK proteins (1–15 out of 32 profiles). Order/disorder plots (VSL2B scores, >0.5 = disordered) of T. gondii (blue) versus P. falciparum (red) proteins that display matching topology protein pairs. Topology profiles for the first fifteen (1–15 out of 32 profiles) drCDC-UNK protein pairs with matching topology are shown. Order/disorder curves for drCDC-UNK protein pairs were aligned by best-fit methods, independent of order/disorder, and ordered by increasing protein length. Note that the set of matching orthologs includes examples of proteins that are nearly fully ordered (<0.5 score) as well as those that are nearly completely disordered (>0.5). (TIF) [file pone.0097625.s002.tif]

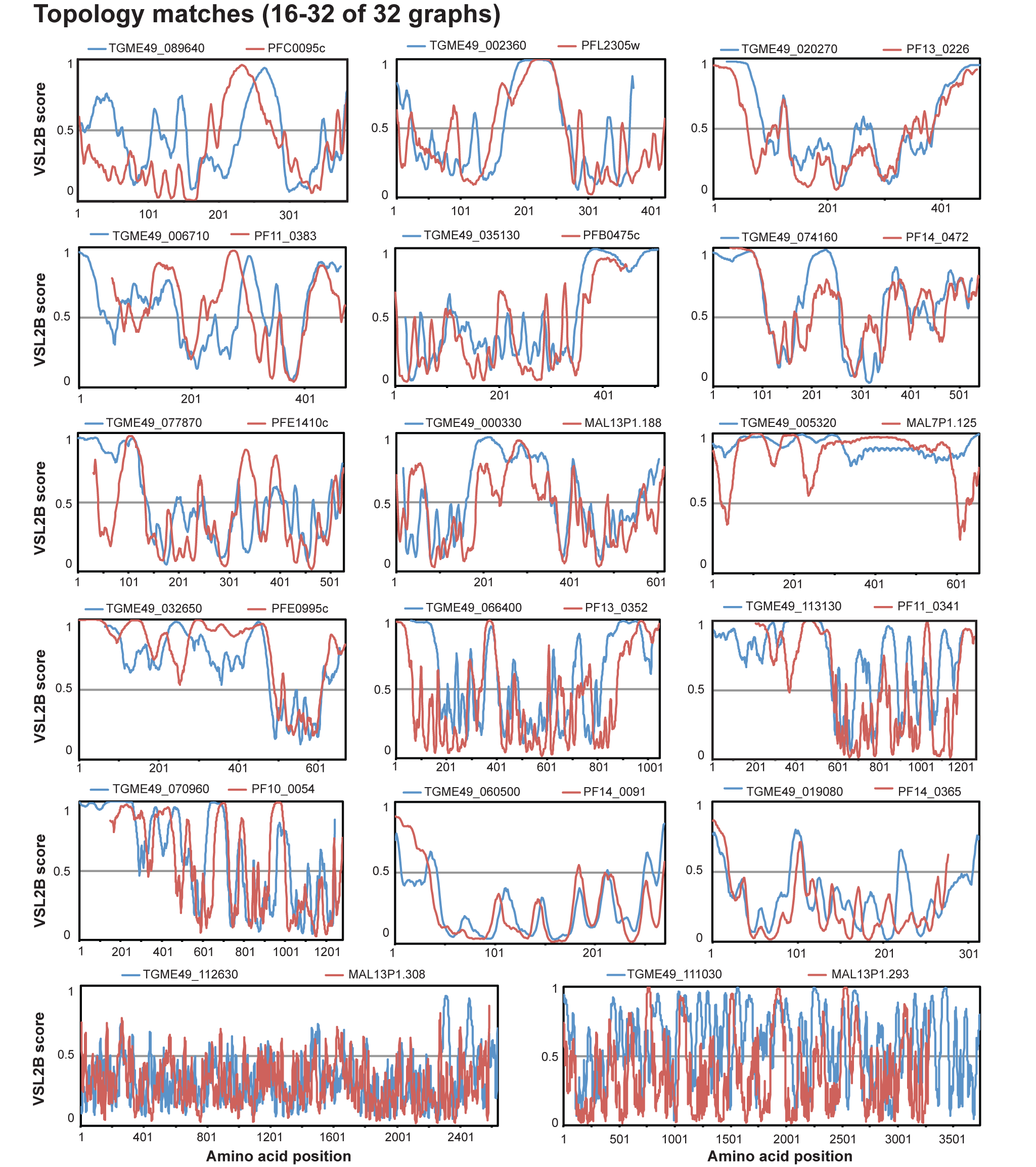

Supplement: Figure S3 — Topology matches for drCDC-UNK proteins (16–32 of 32 profiles). Order/disorder plots (VSL2B scores, >0.5 = disordered) of T. gondii (blue) versus P. falciparum (red) proteins that display matching topology protein pairs. Topology profiles for the next seventeen (16–32 out of 32 profiles) drCDC-UNK protein pairs with matching topology are shown. Order/disorder curves for drCDC-UNK protein pairs were aligned by best-fit methods, independent of order/disorder, and ordered by increasing protein length. The x-axis for longer proteins was extended to better display the protein secondary structure. Note that the set of matching orthologs includes examples of proteins that are nearly fully ordered (<0.5 score) as well as those that are nearly completely disordered (>0.5). (TIF) [file pone.0097625.s003.tif]

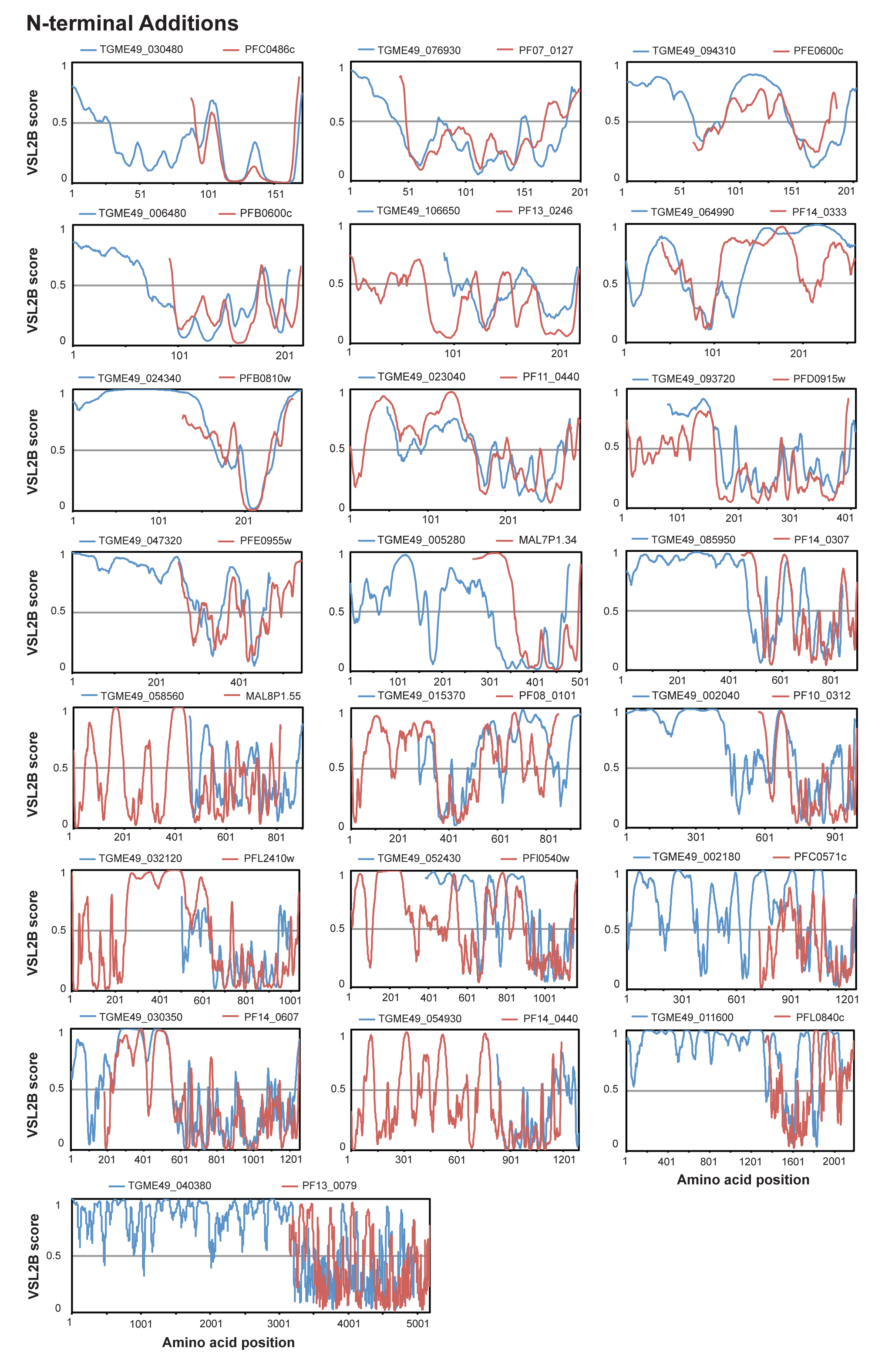

Supplement: Figure S4 — Topology additions of N-terminal domains for drCDC-UNK proteins. Order/disorder plots (VSL2B scores, >0.5 = disordered) of T. gondii (blue) versus P. falciparum (red) proteins that display additions of loops or tails for the protein pairs. Order/disorder curves for drCDC-UNK protein pairs were aligned by best-fit methods, independent of order/disorder, and ordered by increasing protein length. The x-axis for longer proteins was extended to better display the protein secondary structure. Topology profiles of drCDC-UNK proteins with N-terminal domain additions in either species in most cases the domain addition was highly disordered. (TIF) [file pone.0097625.s004.tif]

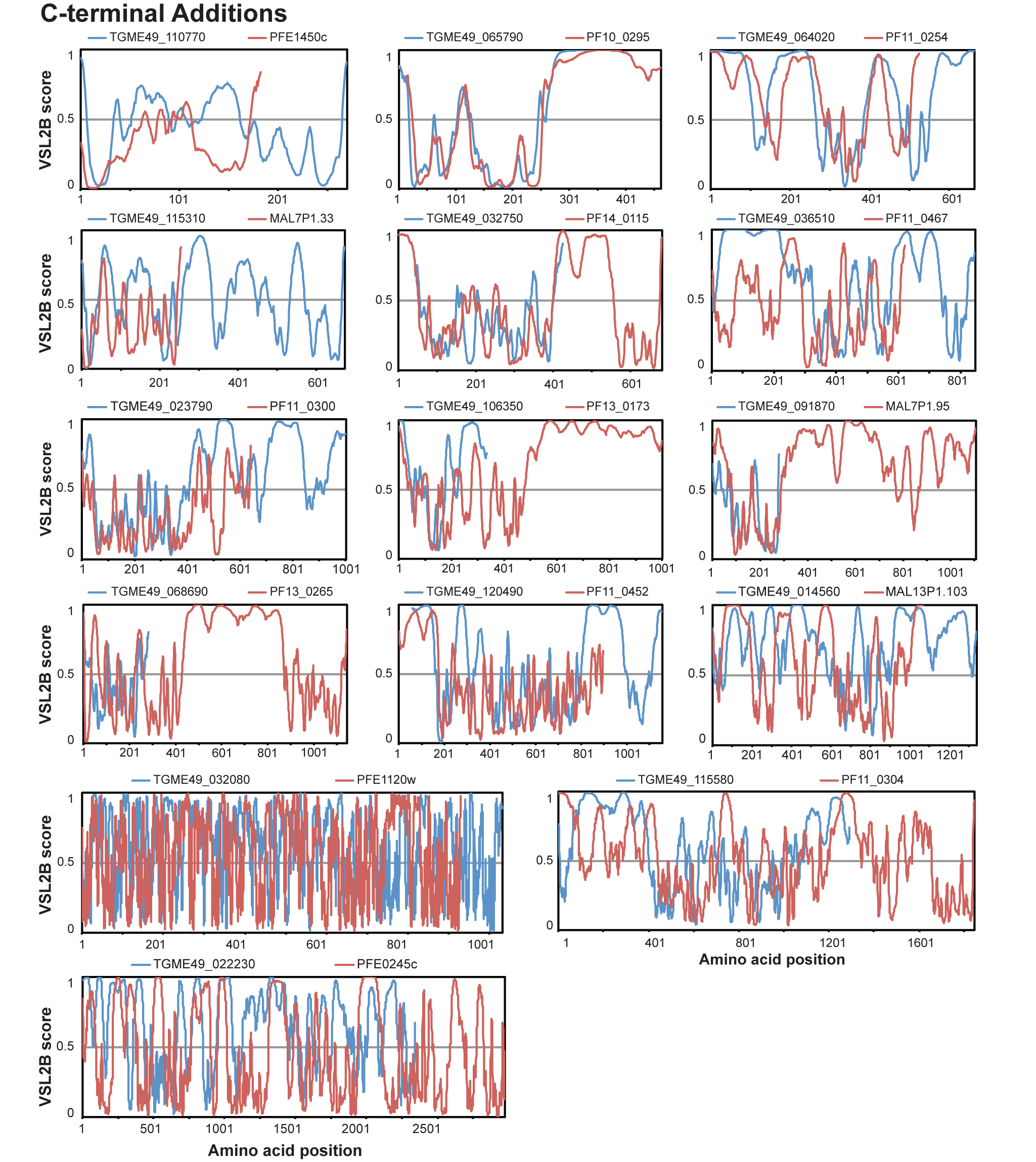

Supplement: Figure S5 — Topology additions of C-terminal tails for drCDC-UNK proteins. Order/disorder plots (VSL2B scores, >0.5 = disordered) of T. gondii (blue) versus P. falciparum (red) proteins that display additions of loops or tails for the protein pairs. Order/disorder curves for drCDC-UNK protein pairs were aligned by best-fit methods, independent of order/disorder, and ordered by increasing protein length. The x-axis for longer proteins was extended to better display the protein secondary structure. Unique C-terminal additions were observed for 15 drCDC-UNK pairs. Similar to N-terminal additions, the C-terminal extension was species specific and disordered. (TIF) [file pone.0097625.s005.tif]

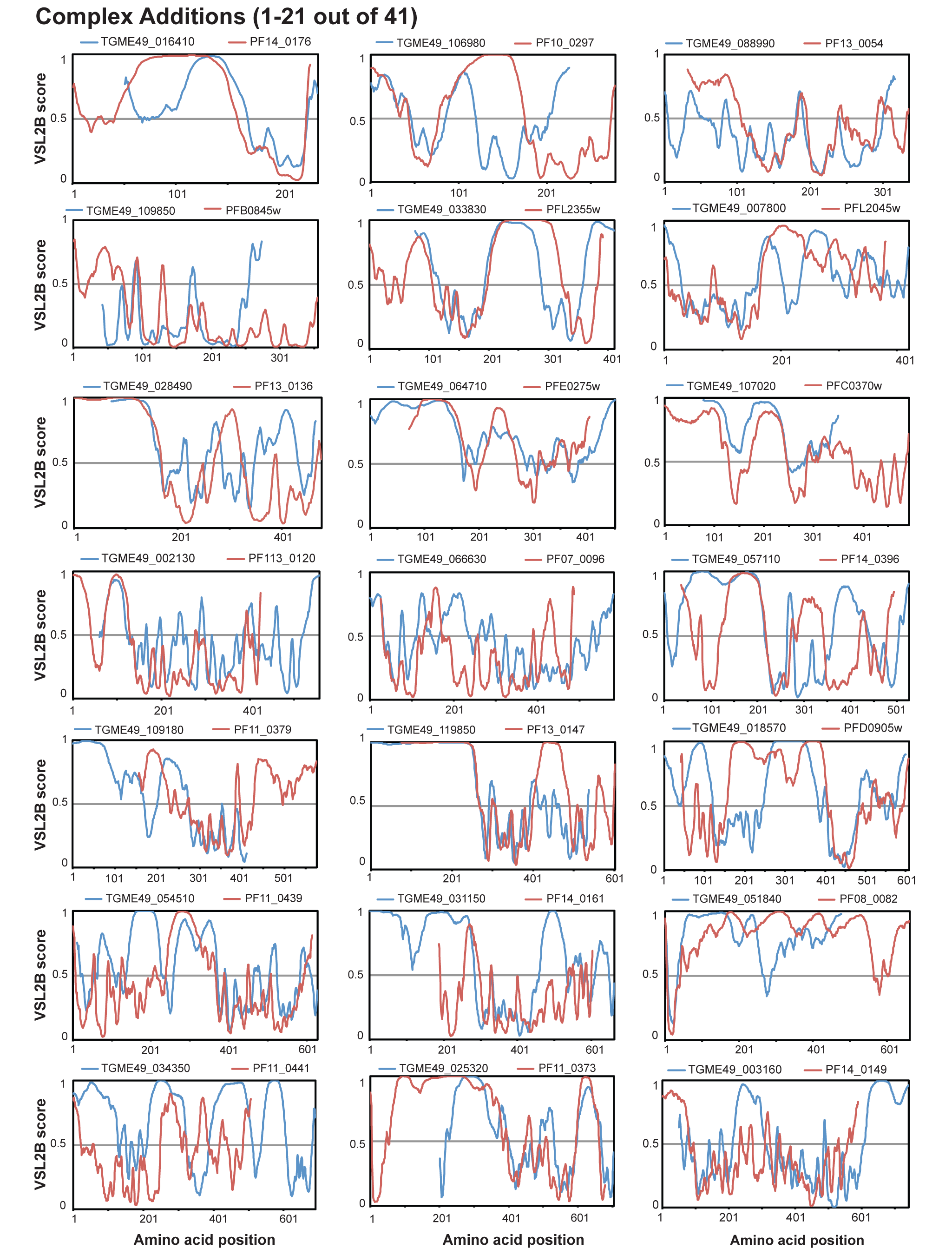

Supplement: Figure S6 — Topology additions of complex additions of tails and loops in drCDC-UNK proteins (1–21 of 41 profiles). Order/disorder plots (VSL2B scores, >0.5 = disordered) of T. gondii (blue) versus P. falciparum (red) proteins that display complex additions were observed in 41 drCDC-UNK pairs and included combinations of N and C-terminal tails or loops. The first 21 profiles of complex additions are shown here. Order/disorder curves for drCDC-UNK protein pairs were aligned by best-fit methods, independent of order/disorder, and ordered by increasing protein length. (TIF) [file pone.0097625.s006.tif]

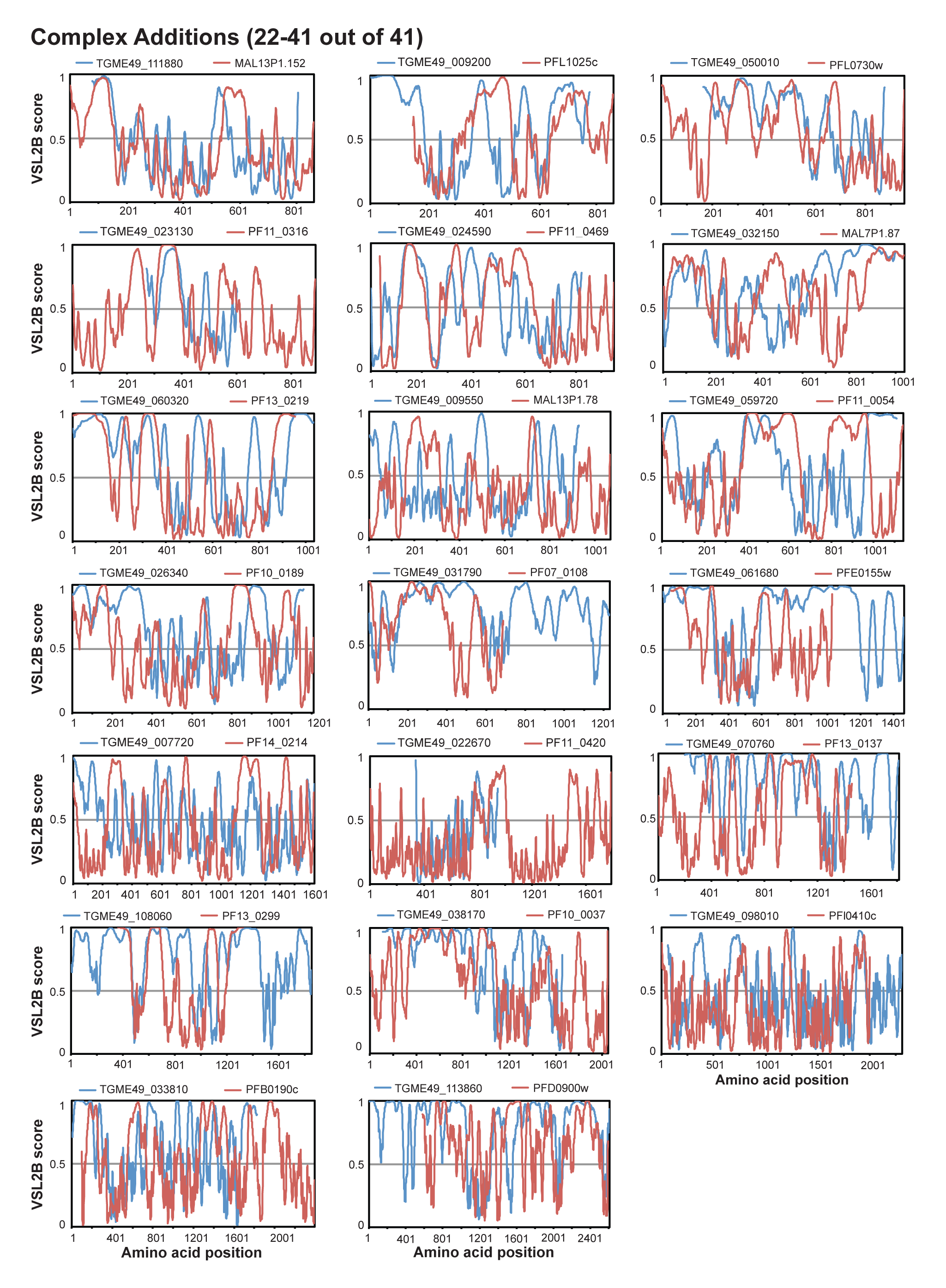

Supplement: Figure S7 — Topology additions of complex additions of tails and loops in drCDC-UNK proteins (22–41 of 41 profiles). Order/disorder plots (VSL2B scores, >0.5 = disordered) of T. gondii (blue) versus P. falciparum (red) proteins that display additions of loops or tails for complex additions were observed in 41 drCDC-UNK pairs and included combinations of N and C-terminal tails or loops. The next 20 profiles of complex additions are shown here. Order/disorder curves for drCDC-UNK protein pairs were aligned by best-fit methods, independent of order/disorder, and ordered by increasing protein length. The x-axis for longer proteins was extended to better display the protein secondary structure. (TIF) [file pone.0097625.s007.tif]

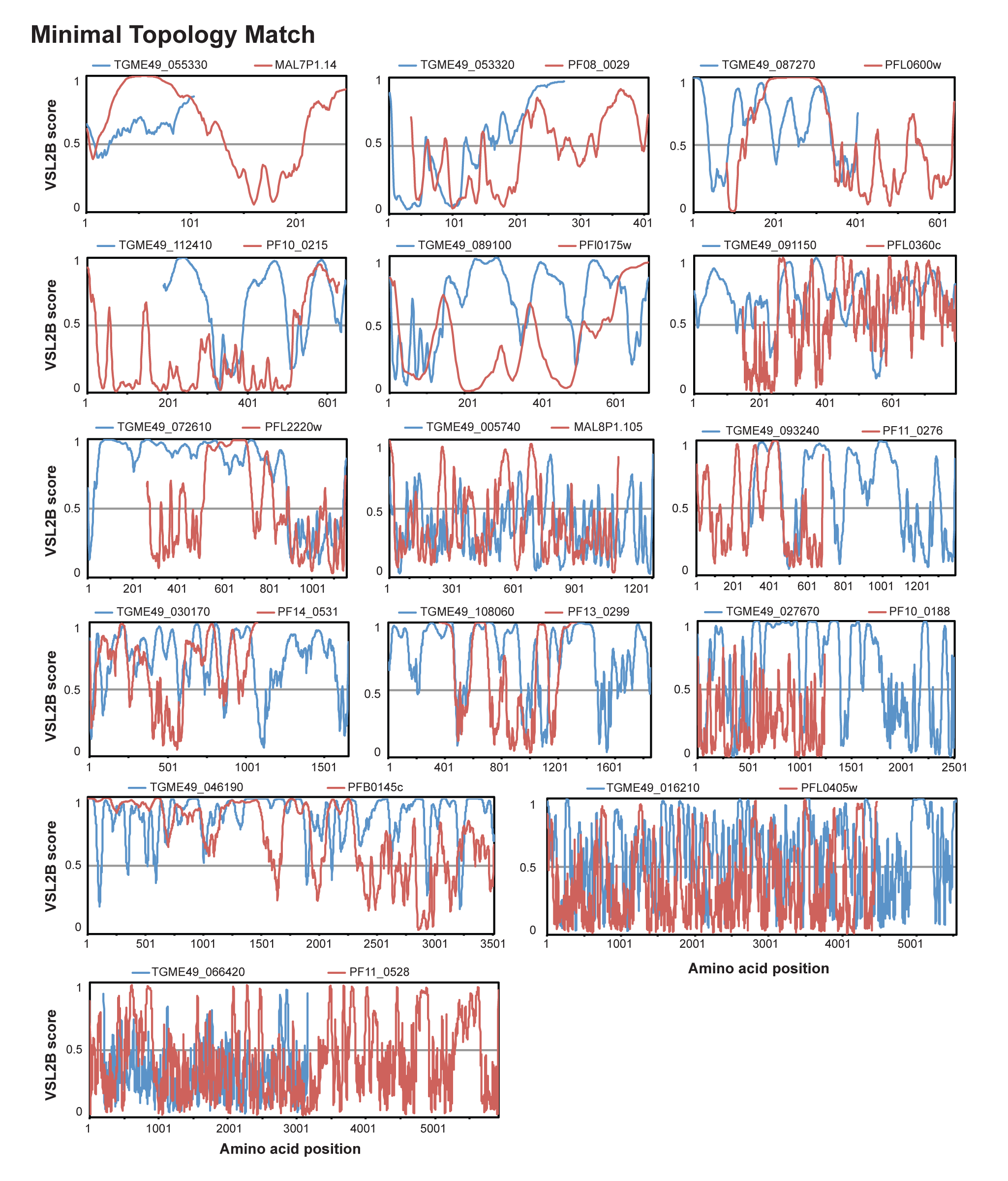

Supplement: Figure S8 — Minimal topology matches for drCDC-UNK proteins. Order/disorder plots (VSL2B scores, >0.5 = disordered) of 15 T. gondii (blue) versus P. falciparum (red) proteins that display minimal topology matched protein pairs. Order/disorder curves for drCDC-UNK protein pairs were aligned by best-fit methods, independent of order/disorder, and ordered by increasing protein length. The x-axis for longer proteins was extended to better display the protein secondary structure. (TIF) [file pone.0097625.s008.tif]

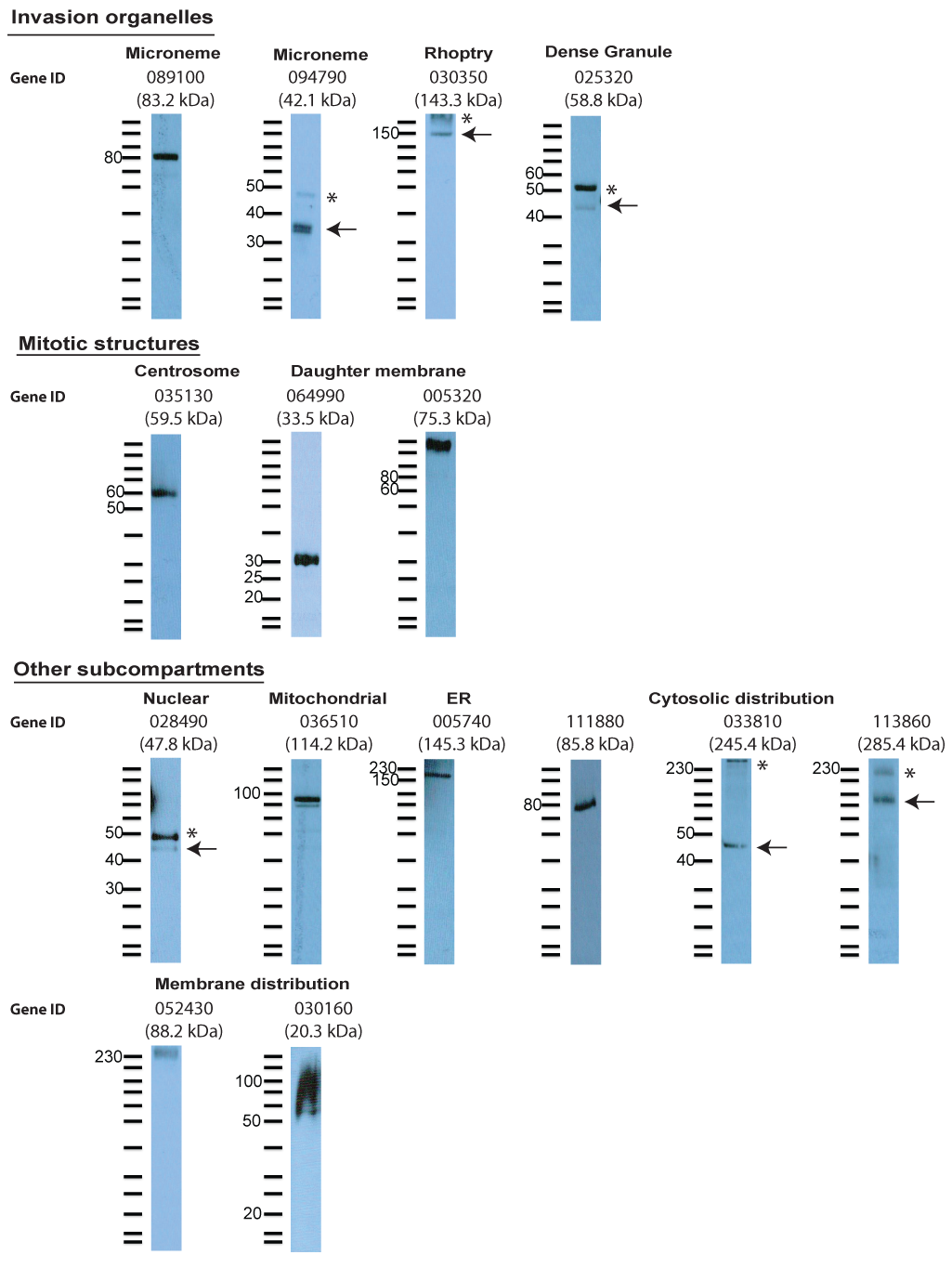

Supplement: Figure S9 — Western analysis of epitope tagged T. gondii drCDC-UNK proteins. Western blot analysis of protein extracts from T. gondii drCDC-UNK tagged strains in Fig. 5 were detected by anti-HA antibody to verify endogenous tagging. Gene IDs correspond to ToxoDB (http://www.toxodb.org/toxo/) assignment for the Type II ME49 strain and are indicated above each western blot image omitting the common “TGME49_” pre-label. The protein marker to the left of each western blot is shown in kDa. Predicted protein sizes from ToxoDB are shown below each gene ID in parenthesis and include the additional 4.4 kDa for the triple HA tag. Asterisks indicate the full-length protein detected by western blot and arrows indicate possible cleaved or degradation products. The analysis showed that 12 out of 16 HA-tagged proteins migrated on Western blots with a mass close to the annotated protein size. Three predicted membrane proteins (TGME49_005320, TGME49_052430 and TGME49_030160) showed anomalous protein sizes, while the protein encoded by TGME49_041000, could not be detected by western analysis. PCR analysis to verify the correct gene knock-in of the 3xHA tag into the appropriate gene locus for these four exceptions are shown in Fig. S10. (TIF) [file pone.0097625.s009.tif]

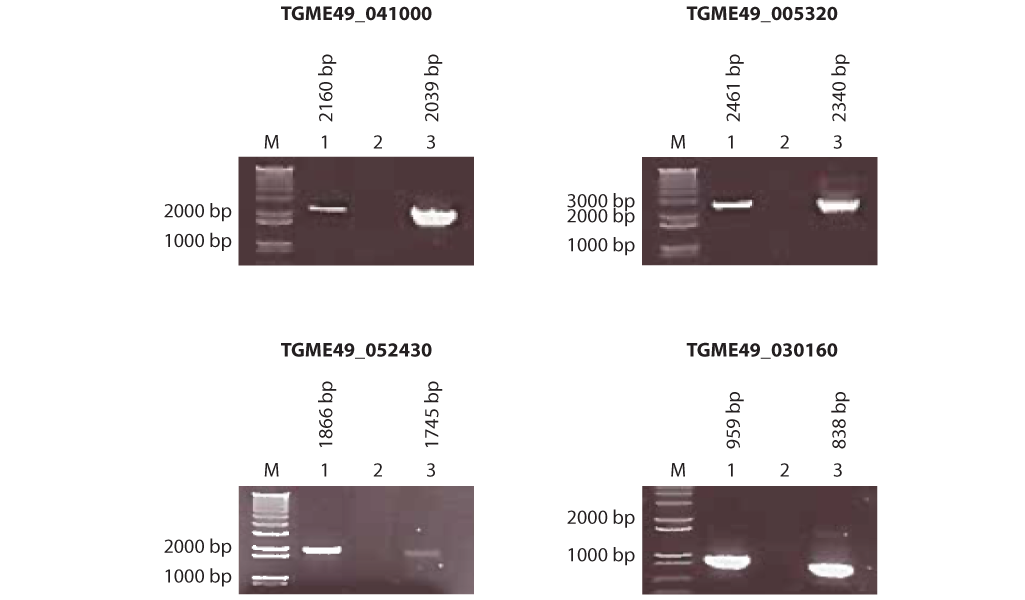

Supplement: Figure S10 — PCR analysis of 3xHA knock-in into the genetic locus of TGME49_041000, TGME49_005320, TGME49_052430 and TGME49_030160. PCR analysis was used to verify tagging of the gene at the correct locus using primers from Dataset S3 for the four drCDC-UNK proteins that failed western blot analysis (Fig. S9). For each of the four PCRs lane 1 shows the genomic DNA (gDNA) from the corresponding endogenously tagged strain amplified with a forward verification primer and a common reverse primer specific for the 3xHA cassette insertion. Lane 2 shows the PCR results from parent strain (RH) gDNA with the forward verification primer and the common 3xHA reverse primer (negative control). Lane 3 shows the PCR result confirming that parent strain gDNA contains the native gene locus; the forward verification primer was combined with a reverse primer binding to native gene sequence that is downstream of the gene knock-in site (present only in the parent gDNA). M = marker and the size of each PCR product is shown above the corresponding lane in base pairs (bp). The analysis demonstrates that all four genes were tagged at the correct locus as evidence by the correct size PCR product for each tagged strain that is larger than the native DNA fragment produced from the native locus present in the parent gDNA. (TIF) [file pone.0097625.s010.tif]

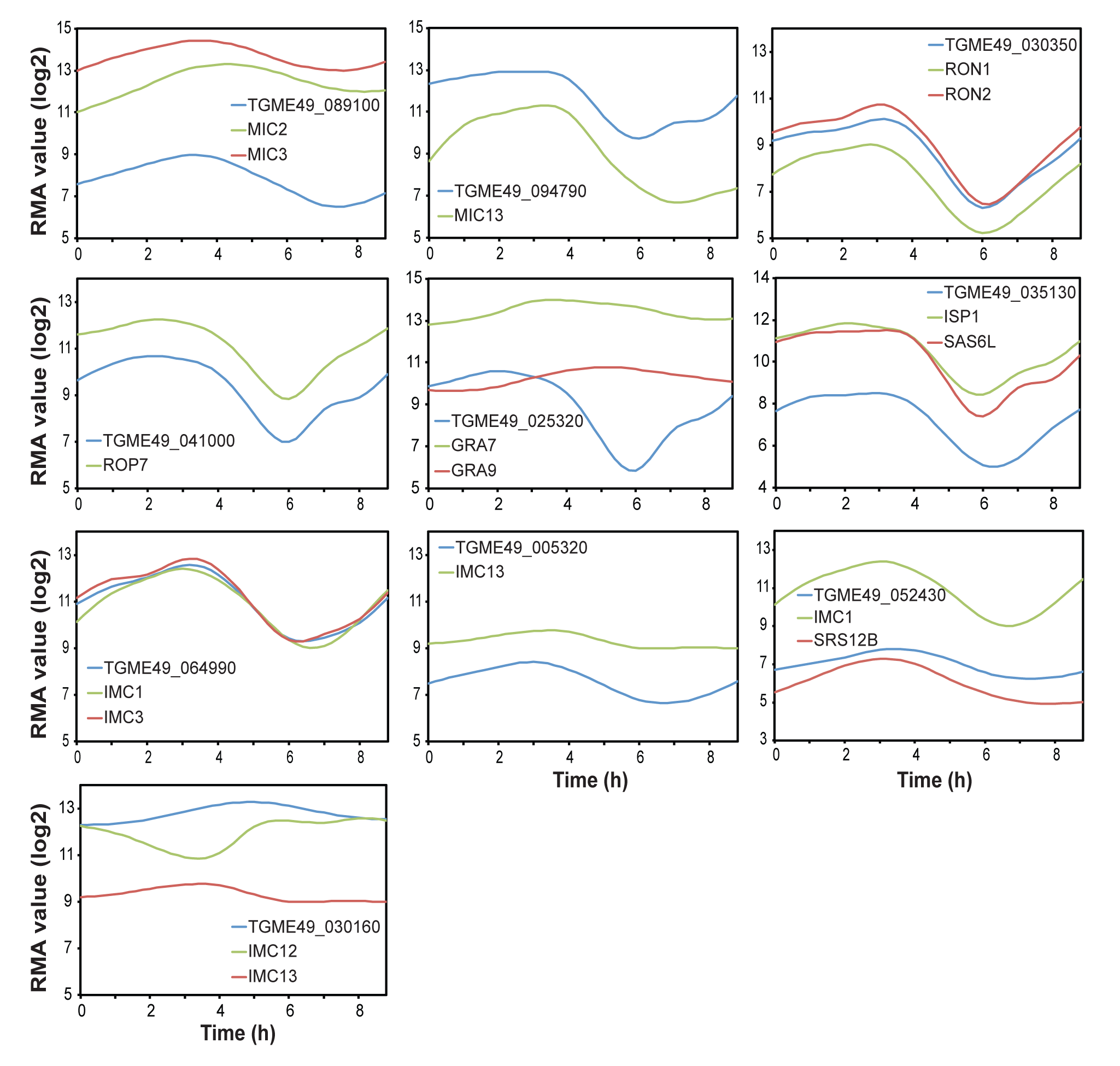

Supplement: Figure S11 — Co-expression graphs for selected drCDC-UNK mRNAs. The mRNA expression [10] for ten T. gondii drCDC-UNK genes (blue line) endogenously tagged in Fig. 5 was compared to previously characterized T. gondii genes (green and red lines) that show a conserved timing of mRNA expression among genes that code for proteins with similar functions. See Behnke et al. 2010 and ToxoDB for cell cycle data for all genes listed and gene IDs for the characterized genes (green and red lines) used to show conserved timing of mRNA peak expression. Plots of mRNA expression profiles show mRNA expression levels (RMA value, y-axis) throughout the 8.75 h synchronized lifecycle of T. gondii tachyzoites (in hours, x-axis). Due to the method of synchrony that arrests T. gondii RHTK+ tachyzoites in late G1/early-S transition (thymidine block and release) [4], [10], we partitioned the cell cycle into early G1 = 4.6–6.5 h, late G1 = 6.6–8.75 h, and S/M/C = 0–4.5 h. (TIF) [file pone.0097625.s011.tif]
